# TrkB inhibition of DJ-1 degradation promotes the growth and maintenance of cancer stem cell characteristics in hepatocellular carcinoma

**DOI:** 10.1007/s00018-023-04960-z

**Published:** 2023-09-25

**Authors:** Min Soo Kim, Won Sung Lee, Wook Jin

**Affiliations:** 1 Laboratory of Molecular Disease and Cell Regulation, Department of Biochemistry, School of Medicine, Gachon University, Incheon, 21999 Republic of Korea; 2 Department of Health Sciences and Technology, GAIHST, Gachon University, Incheon, 21999 Korea

**Keywords:** TrkB, Hepatocellular carcinoma, Epithelial–mesenchymal transition (EMT), DJ-1, Ubiquitination, Chemoresistance, Acquisition of cancer stem cells

## Abstract

Although TrkB may be associated with the pathogenesis of various cancer by upregulation, how upregulation of TrkB led to tumor progression in hepatocellular carcinoma (HCC) and the signaling mechanisms by which TrkB induces motility, invasion, metastasis, drug resistance, and acquisition of self-renewal traits has remained unclear. Here, we demonstrated that TrkB was significantly upregulated in highly metastatic HCC cells and HCC patients. Also, the increased TrkB levels were significantly correlated with tumor stages and poor survival of HCC patients. Furthermore, the upregulated TrkB expression enhances the metastatic ability of HCC cells through reduced anoikis sensitivity, induced migration, and colony formation. Most strikingly, TrkB markedly enhances the activation of STAT3 by preventing DJ-1 degradation through the formation of the TrkB/DJ-1 complex. This signaling mechanism is responsible for triggering cellular traits of highly aggressive HCC. The activation of the EMT program of HCC via increasing DJ-1 stability by TrkB induces the gain of cancer stem cell states and chemoresistance via the upregulation of stem cells cell markers and ABC transporters. Also, TrkB-mediated inhibition of DJ-1 degradation promotes tumor formation and metastasizes to other organs in vivo. Our observations illustrate that TrkB is a prognostic and therapeutic targeting in promoting aggressiveness and metastasis of HCC.

**Supplementary Information:**

The online version contains supplementary material available at 10.1007/s00018-023-04960-z.

## Background

Several studies suggest that tropomyosin-related kinase (Trk) B receptor, tyrosine kinase, plays a crucial role in neurophysiological processes of the central nervous system, including survival and development [1], and its expression was correlated with neurodegenerative diseases such as Parkinson’s disease [2] and Alzheimer’s disease [3, 4]. In addition, TrkB is implicated in cancer development. TrkB was upregulated in various cancer types, and this upregulation of TrkB was correlated with progression and poor survival of cancers [5, 6], suggesting that upregulation of TrkB might associate with the pathogenesis of various cancer.

A small body of research revealed the TrkB-mediated signaling mechanisms, which induce tumorigenicity and metastasis. Since TrkB inhibits cell death via AKT activation [7, 8], some signal mechanisms regulated by TrkB have been reported in cancers. TrkB leads to colon cancer metastasis through the upregulation of Runx3, Keap1 [6], and suppression of etoposide-mediated upregulation of p53 and PUMA [9]. Also, TrkB induces cell survival of colon and breast cancer through the activation of ERK [10] or JAK2/STAT3 signaling pathways [11]. Moreover, BDNF-mediated activation of TrkB promotes brain metastases through the formation of the TrkB/Her2 complex [12] and confers neuroblastoma cells against chemoresistance by induction of hypoxia-inducible factor 1α (HIF-1α) [13]. In addition, STAT3 activation-induced BDNF increases the proliferation of NSCLC by prolonged TrkB activation [14].

Independent of these studies, we and others previously showed that TrkB, as the diagnostic and therapeutic target, enhances the growth of HepG2 cells through inhibition of DNA methylation [15, 16]. Although TrkB may be associated with malignancy of various cancer, it is not fully understood how TrkB is involved in cancer pathogenesis, acquisition, and maintenance of cancer stem cell (CSC) state, and chemoresistance in hepatocellular carcinoma (HCC).

In the present study, we find that TrkB directly induces the acquisition of mesenchymal trait and chemoresistance through activation of cellular traits, including the EMT program associated with aggressiveness. Also, TrkB specifically interacted with DJ-1, thereby increasing DJ-1 stabilization by blocking the ubiquitin-induced DJ-1 degradation, and subsequently upregulated DJ-1 induces STAT3 activation to activate invasion, metastasis, and chemoresistance.

## Methods

### Cell cultures

The human HCC cell lines (PLC/PRF/5, Chang, HepG2, HUH7, SNU182, SNU354, SNU368, SNU387, SNU449, and SNU761) were cultured in high-glucose DMEM (11965-092; Gibco, Thermo Fisher Scientific, Inc., Waltham, MA, USA) supplemented with 10% FBS (10099141; Gibco) and penicillin–streptomycin (15140-122; Gibco) at 37 °C under a 5% CO2 condition [15, 17]. For Adriamycin and etoposide treatments, SNU387 control-shRNA or TrkB-shRNA cells were exposed to Adriamycin and etoposide final concentration of 10 μg/ml for indicated times. Adriamycin and etoposide were obtained from Sigma.

### Antibodies

The main primary antibodies used in this study were as follows: anti-E-cadherin (#3195S), anti-fibronectin (#26836S), anti-N-cadherin (#13116S), anti-alpha-catenin (#2131S), and anti-vimentin (#5741S), anti-STAT3 (#9139S), anti-Snail (#3879S), and anti-phospho-STAT3 (#9145S) were from Cell Signaling Technology; anti-TrkB (ab18987), anti-Twist-1 (ab50887), and anti-DJ-1 (ab18257) were from Abcam; anti-HA (sc-7392) and anti-GFP (sc-9996) were from Santa Cruz Biotechnology; anti-V5 (MA5-15,253) was from Life Technologies; and anti-β-actin (A1978) and anti-Flag (F3165) were from Sigma (Table S2).

### In silico analysis of clinical microarray data

TrkB, Twist-1, Snail, CD133, CD117, CD90, and CK19 expression signatures from HCC patients were extracted and averaged using the GSE14323 [18], GSE17967 [19], GSE5975 [20], and TCGA [21] datasets, and then in silico analysis was performed. The boxplot graphs were plotted with gene expression using GraphPad Prism v 5.0 (GraphPad Software, Inc.), and *P* < 0.05 was considered statistically significant.

### Western blot, immunoprecipitation, immunofluorescence, immunohistochemistry analysis

All analyses were performed as described previously [22, 23]. Protein lysate from cells and cochlear sensory epithelia for each group was prepared in RIPA buffer (R2002; Biosesang) with the addition of protease inhibitor (11697498001; Roche), followed by placement on ice for 30 min, being shaken every 10 min. After the centrifugation at 12,000 rpm at 4 °C for 10 min, the supernatants were denatured and loaded on 10% SDS-PAGE gel. Proteins were then transferred in an ice bath to a polyvinylidene difluoride membrane (Immobilon-P, IPVH00010; Millipore, Schaffhausen, Switzerland). After blocking with PBST buffer containing 5% milk for 1 h, the membrane was incubated with primary antibodies overnight at 4 °C, followed by secondary antibody.

For transient transfection, 293T, SNU387 shTrkB, and PLC/PRF/5 cells were seeded 24 h before transfection in the 6-well plates. Immediately before transfection, regular medium was replaced with medium containing 1% FBS. Transfections were performed using Lipofectamine 2000 (Invitrogen) transfection reagent according to the manufacturer’s recommendations. The plasmids used were pEF6-v5-TrkB, pGFP-DJ-1, and pCMV-flag-STAT3; each concentration was 3ug/ml. The immunoprecipitation (IP) experiment was performed to determine if there was an interaction between STAT3 and DJ-1 proteins and between TrkB and DJ-1 proteins. 293T cells were seeded in the 6-well plates overnight. Then cells were scraped in the presence of RIPA lysis buffer. After the centrifugation at 12,000 rpm at 4 °C for 10 min, the cellular proteins were incubated with anti-GFP (1:100; sc-9996), anti-Flag (1:100; F3165), anti-DJ-1 (1:1000; ab18257), and anti-V5 (1:100; MA5-15253) primary antibody of volume of 2µL, overnight at 4 °C on a rocking platform. The next day, 50 µL Protein A Agarose (sc-2001) and cellular proteins (primary antibody incubated) were mixed and incubated for 1 h. The cells were centrifuged at 3000 rpm at 4◦C and washed twice, followed by Western blotting with the primary antibodies used for the IP experiments. For immunofluorescence, 1 × 10^4^ cells were seeded on Labtek II chamber slide (Nunc), and cells were fixed with 4% paraformaldehyde for 15 min and permeabilized in a 0.1% Triton X-100 solution at room temperature for 1 h. Finally, cells were incubated with a primary antibody overnight at 4 °C and incubated with a secondary antibody for 1 h. The image was observed under a fluorescence microscope at 488 and 594 nm wavelengths. Micrographs were captured using confocal software (Zeiss).

For immunohistochemistry analysis, normal, HCC, or metastatic HCC tissues from a tissue microarray slide were purchased from Super Bio Chips (CSA5). The sections were deparaffinized twice with xylene (for 20 min each), then rehydrated twice with 100% alcohol (for 5 min each), then rehydrated with 75% alcohol (for 5 min each), and rinsed with water. The sections were deparaffinized and rehydrated and, after antigen retrieval and blocking, incubated with the primary antibody. The primary antibody includes anti-TrkB (1:100; ab134155). The section was incubated with peroxidase-labeled secondary antibody and stained by DAB reagent. Imaging of vessels was observed under Zeiss inverted microscopy.

### Colony formation, Anoikis, Matrigel invasion, and wound healing assays

Assays were performed as previously described [24, 25]. For the colony formation assay, 1 × 10^4^ cells were seeded in the 6-well plates and cultured for 14 days. Then cell colonies were fixed in formaldehyde and stained with 1% crystal violet. For the anoikis assay, 1 × 10^4^ cells were seeded in the 6-well plates and were counted after 14 days. For the cell migration and invasion assay, 1 × 10^3^ cells were seeded into the 24-well invasion chambers with an 8.0 µm polyethylene terephthalate membrane (354483; Corning). PLC/PRF/5, PLC/PRF/5-TrkB, SNU387, and SNU387 TrkB-shRNA cells were serum starved overnight and then seeded into the Transwell inserts, and DMEM supplemented with 10% FBS was added to the lower part of the chamber. The cells inside the chamber were carefully removed by a cotton tip that had been moistened with PBS, and the migrated cells were fixed in 4% paraformaldehyde (PFA, 28908, Thermo Fisher Scientific) for 10 min. These migrated cells were stained with 0.5% crystal violet for 30 min. 3–5 fields/filters were counted. All the experiments were performed in triplicates, and the representative results are shown.

For wound healing assay, 1 × 10^5^ cells were seeded into 6-well plate. The gaps between cells at 0 h were created by pipet tip. The cells were then incubated in a medium with 10% FBS, and images were captured at 0 h and 24 h. Then, images were analyzed using Image J software to measure the remaining gap area at each time point. For the spheroid assay, 1 × 10^4^ cells per well were seeded into the Ultra-Low attachment multiple-well plate (Corning) in DMEM/F12 medium supplemented with 20 ng/ml FGF (Sigma), 20 ng/ml EGF (Sigma), B27 (GIBCO). After indicated times (14 days), the cells were visualized via fixed and stained using crystal violet solution (Sigma), and cultured cells were photographed at indicated times.

### Luciferase reporter assay

Luciferase reporter assays were performed as previously described [24]. 1 × 10^4^ cells were seeded in 12-well dishes were transfected using Lipofectamine 2000 (Invitrogen). A total of 0.5 μg pGL3-STAT3 or pGL3-CD133 reporter gene constructs and 0.5 μg of pCMV-β-gal were co-transfected per well. The cell extracts were prepared 48 h after transfection, and the luciferase activity was quantified using the Enhanced Luciferase Assay Kit (BD Biosciences). All experiments were performed in triplicate. The human STAT3 promoter (− 1555/ + 133) (Sequence ID: NM_213662) and human CD133 promoter (− 1313/− 1) (Sequence ID: NM_001145847) were cloned into the pGL3 Vector (Figure S1).

### Reverse transcriptase PCR (RT-PCR) and real-time RT-PCR analysis

RNA preparation, RT-PCR, and Real-time RT-PCR analysis were performed as previously described [11, 24]. RNA was isolated using the RNeasy Micro Kit (QIAGEN), and reverse transcription was performed with QuantiTect Reverse Transcription Kit (QIAGEN). Gene expression was quantified by SYBR Premix Ex Taq Kit (Takara Bio) in CFX96 TouchTM Real-Time PCR Detection System C1000 Thermocycler (BioRad). The primer sequences are listed in the supplemental experimental procedures (Table S1). PCR reactions were performed at 50 °C for 2 min and 95 °C for 2 min, followed by 40 cycles of 95 °C for 15 s and 60 °C for 1 min. Cycle threshold (CT) values for individual reactions were obtained using CFX Manager Software (BioRad). To determine relative gene expression levels, the CT values were normalized to the housekeeping gene GAPDH using the ΔCT method.

### Animal studies

Research involving animals were handled in compliance with protocols approved by the Institutional Animal Care and Use Committee (IACUC) of Gachon University (Approval No. LCDI-2012-0016) and were performed as previously described [11, 24]. For tumorigenicity studies, 1 × 10^6^ cells suspended in PBS were injected subcutaneously into the hind flank regions of female BALB/c Nu/Nu mice (7 weeks old, *n* = 7) and were euthanized and necropsied at 32 days. For the metastases of the lung and liver of mice, the lungs and livers were excised, fixed in 10% formalin, paraffin embedded, and sectioned for analysis.

### Statistical analysis

Each experiment was repeated at least three independent times unless otherwise indicated. Data are presented as the means ± SEM. Statistical analyses of the data were conducted via Student’s *t* test (two-tailed) and ANOVA. *P* < 0.05 or* P* < 0.001 were considered statistically significant.

## Results

### Upregulation of TrkB expression was significantly associated with the pathogenesis of HCC

**Fig. 1:**
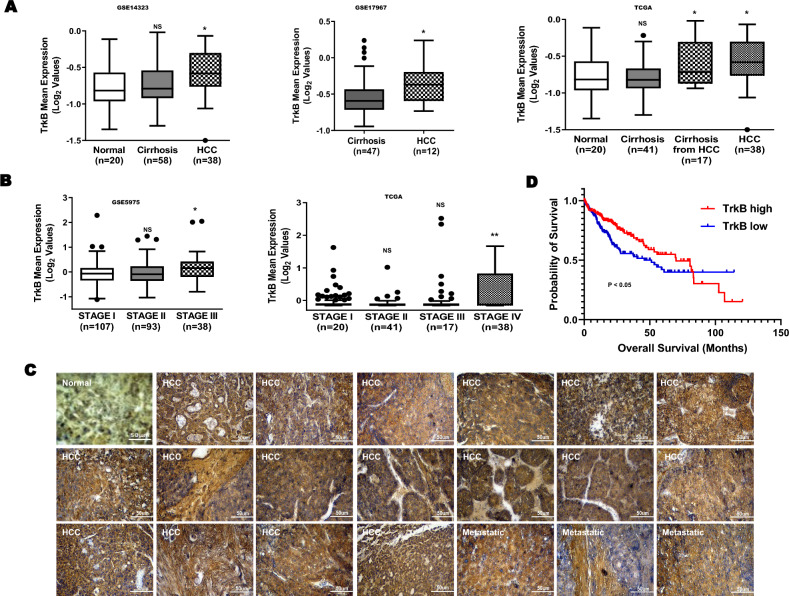
Contribution of TrkB expression to the progression of HCC patients. **a** Box-and-whisker (Tukey) plots of TrkB expression in human HCC patients. The signature of TrkB expression was extracted from the GSE14323, GSE17967, and TCGA datasets and averaged. *P* < 0.05 (*); NS: not significant; *t* test. **b** Box-and-whisker (Tukey) plots of TrkB expression in the stages of human HCC patients. The signature of TrkB expression was extracted from the GSE5957 and TCGA datasets and averaged. *P* < 0.05 (*) or *P* < 0.005 (**); NS: not significant; *t* test and one-way ANOVA. **c** Representative immunohistochemical images of TrkB staining in normal human healthy liver tissue, HCC, and metastatic HCC (magnification: 200 ×). Scale bar = 50 µm. **d** Kaplan–Meier analysis of overall survival of individuals with HCC patients between high or low TrkB expression. The average expression value of TrkB was determined, rank ordered, and then divided into two equal groups. *P* < 0.05 (*); log-rank test

Although the TrkB upregulation is linked to certain types of cancer, the role of TrkB has not been characterized in the pathogenesis of HCC. To characterize whether the differences in the level of TrkB were associated with differences in tumor histology, we first investigated the clinical relevance using public HCC microarray datasets to interrogate the correlation between the expression of TrkB and the progression of HCC. Because TrkB expression has also been shown to be highly expressed in HCC cells, we compared the levels of TrkB expression in patients with cirrhosis and HCC using a clinical study (GSE14323 [18], GSE17967 [19]) from the Gene Expression Omnibus (GEO) and TCGA dataset [21]. HCC patients from GSE14323, GSE17967, and the TCGA dataset of the clinical study showed significantly increased TrkB protein levels relative to normal and patients with cirrhosis (Fig. 1a). Once again, TrkB expression is significantly correlated with the stage of HCC. The level of TrkB expression from the TCGA dataset and GSE5975 [20] was drastically higher in stages III and IV relative to other stages of HCC (Fig. 1b).

We next examined the clinical relevance using normal and tumor tissues from 44 HCC patients. Consistent with the above observations, the level of TrkB expression was drastically upregulated in HCC or metastatic cancer patients relative to healthy tissues (Fig. 1c). Moreover, in our present work, high TrkB expression correlates strongly with the poor survival outcome of HCC patients (Fig. 1d). Thus, our observations suggest that TrkB expression is critical to the pathogenesis of HCC.

### Identification of TrkB as an essential protein for the aggressiveness of hepatocellular carcinoma

**Fig. 2:**
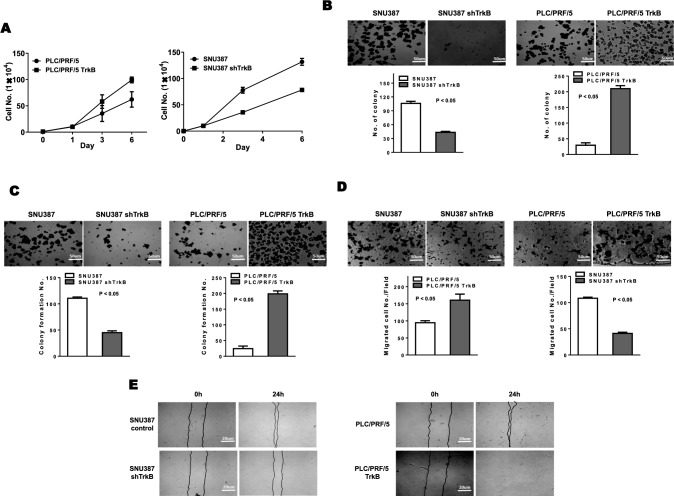
Effect of TrkB expression on the metastatic potential of hepatocellular carcinoma cells. **a** In vitro cell growth analysis of PCL/PRF/5, PCL/PRF/5 TrkB, SNU387 control-shRNA, or SNU387 TrkB-shRNA cells. The data are presented as three independent experiments’ mean ± SEM values. **b** Soft agar assay and bright-phase microscopy images of PCL/PRF/5, PCL/PRF/5 TrkB, SNU387 control-shRNA, or SNU387 TrkB-shRNA cells. The data are presented as three independent experiments’ mean ± SEM values. *P* < 0.05, *t* test. **c** Formation of spheroid colonies and bright-phase microscopy images of PCL/PRF/5, PCL/PRF/5 TrkB, SNU387 control-shRNA, or SNU387 TrkB-shRNA cells. The data are presented as three independent experiments’ mean ± SEM values. *P* < 0.05, *t* test. **d** Migration assay and bright-phase microscopy images of PCL/PRF/5, PCL/PRF/5 TrkB, SNU387 control-shRNA, or SNU387 TrkB-shRNA cells. The data are presented as three independent experiments’ mean ± SEM values. *P* < 0.05, *t* test. e Wound healing assay and bright-phase microscopy images of PCL/PRF/5, PCL/PRF/5 TrkB, SNU387 control-shRNA, or SNU387 TrkB-shRNA cells

Previous studies demonstrated that PLC/PRF/5 cells had the features of epithelial cells. In contrast, SNU387 cells were classified as mesenchymal cells based on the expression of epithelial and mesenchymal markers. Also, mesenchymal HCC cells significantly correlated with increased expression of genes, which involved in the metastasis of HCC. Moreover, SUN387 cells exhibit significantly reduced sorafenib, erlotinib, gefitinib, and cetuximab sensitivity [26, 27]. These results demonstrated the connection between the metastasis and tumorigenesis of HCC cells and TrkB expression. To address this question, we sought to compare the ability of the TrkB knockdown cells and TrkB overexpression cells to metastasize and proliferate within a distant organ. To assess the functional link between TrkB expression and the metastatic potential of HCC, we first observed TrkB expression in HCC cells. We found that TrkB expression markedly increased in HUH7, SNU182, SNU354, SNU368, and SNU387 cells but not increased in other HCC cells (SNU449 and SNU761) relative to Chang and HepG2 cells, which is known not to express TrkB (Figure S2). We next observed whether TrkB regulates the proliferation of HCC. To test this hypothesis, we prepared PLC/PRF/5-TrkB by a vector that expresses V5-tagged TrkB and SNU387 TrkB-shRNA cells in which expression of the endogenous TrkB was depleted by short hairpin RNA (data not shown). As expected, relative to the parental PLC/PRF/5 cells, TrkB expression increased the proliferation rate in PLC/PRF5 cells, but TrkB knockdown in SNU387 cells significantly reduced the growth rate relative to the SNU387 cells (Fig. 2A). To examine for another hallmark of cancer cells, we examined whether TrkB induces colony-forming ability of HCC cells. PLC/PRF/5-TrkB and SNU387 control-shRNA cells were 2.4- and 7.1-fold enriched in colony-forming ability relative to PLC/PRF/5 and SNU387 TrkB-shRNA cells, respectively (Fig. 2b). We next conducted an anoikis assay to test the effects of TrkB expression on an acquisition of anchorage independence and observed a similar elevation of around 2.5- and 8.2-fold in sphere formation in PLC/PRF/5-TrkB and SNU387 control-shRNA cells compared to PLC/PRF/5 and SNU387 TrkB-shRNA cells, respectively (Fig. 2c).

We also performed the cell migration and wound healing assays, indicating that the loss of TrkB expression in HCC cells markedly attenuated the migration of cancer cells. During cell migration, we observed an almost 1.7- and 2.6-fold reduction in migration efficiency of PLC/PRF/5 and SNU387 TrkB-shRNA cells compared to PLC/PRF/5-TrkB and SNU387 control-shRNA cells (Fig. 2d, e). These observations revealed that TrkB expression might enhance and maintain the malignant traits in HCC cells to seed new tumors in distant organs.

### TrkB induces activation of STAT3 by increasing the stabilization of DJ-1

The upregulation of DJ-1 leads to proliferation, aggressiveness, and poor survival of HCC patients by inducing the level of phospho-STAT3 expression [28]. Also, DJ-1 induces astrogliosis via enhancing STAT3 activation [29]. However, it remains poorly understood about the precise molecular mechanism of DJ-1-derived STAT3 activation. In addition, we previously noted that TrkB activates the IL6/JAK2/STAT3 signaling pathway in breast cancer via the formation of the TrkB/JAK2 complex and then induces the EMT program by upregulating Twist-1 [11], suggesting that DJ-1 and TrkB may orchestrate to activate the STAT3 signaling pathway. Therefore, these previous reports, including our study, led us to investigate that TrkB might activate STAT3 activation by regulating the functional role of DJ-1 via a functional link with DJ-1 in HCC.

**Fig. 3:**
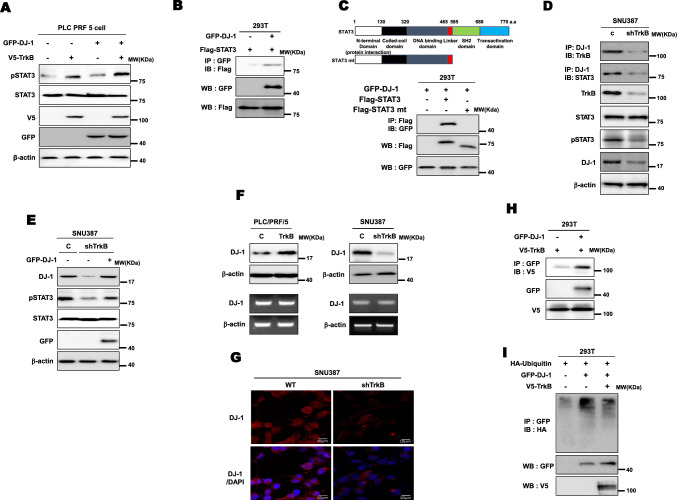
TrkB promotes the activation of STAT3 by inhibiting the degradation of DJ-1. **a** Immunoblot analysis of phospho-STAT3, STAT3, TrkB, and DJ-1in PLC/PRF/5 after transfected with plasmids used were pEF6-v5-TrkB and pGFP-DJ-1, and each concentration was 3ug/ml. β-actin used as a loading control. **b** Identification of complex formation of DJ-1/STAT3 by immunoprecipitation. **c** Identification of the STAT3 region that interacted with DJ-1. **d** Immunoblot analysis of the cell lysates and immunoprecipitation derived from SNU387 and SNU387 shTrkB cells. **e** Immunoblot analysis of phospho-STAT3, STAT3, and DJ-1 in SNU387 TrkB-shRNA cells after transfected with DJ-1. β-actin used as a loading control. **f** Immunoblot and RT-PCR analysis of DJ-1 in PLC/PRF/5, PLC/PRF/5 TrkB, SNU387 control-shRNA, and SNU387 TrkB-shRNA cells. β-actin used as a loading control. **g** Immunofluorescence images of DJ-1 in SNU387 control-shRNA and SNU387 TrkB-shRNA cells. Scale bar = 10 µm. **h** Identification of complex formation of DJ-1/TrkB by immunoprecipitation. **i** Immunoblot analysis of whole-cell lysates and immunoprecipitation derived from 293T cells transfected with the ubiquitin, GFP-DJ-1, and V5-TrkB constructs, as indicated

To address this question, we examined the protein levels of phosphorylated STAT3 and STAT3 in the presence of DJ-1 or TrkB. As a result, we could detect phosphorylated STAT3 levels markedly increased in the expression of both DJ-1 and TrkB, relative to TrkB or DJ-1, respectively. Interestingly, these STAT3 activations are more induced in the presence of TrkB than DJ-1 (Fig. 3a). In addition, we confirmed that STAT3 expression levels using qRT-PCR and luciferase reporter analysis were not different with or without DJ-1 or TrkB (Figure S3A and S3B). These results indicate that DJ-1 or TrkB may activate STAT3 by forming the DJ-1/STAT3 or TrkB/STAT3 complex. We next confirmed this possibility by immunoprecipitation analysis. Interestingly, DJ-1 directly binds to STAT3 to regulate STAT3 activation (Figs. 3b, d and S4A), but TrkB does not interact with STAT3 (Figure S4B), and the C-terminal 86 amino acids of STAT3 containing the SH2 and transactivation domains required for its interaction with DJ-1 (Figs. 3c and S4C). However, it is still unclear how TrkB is involved in DJ-1-mediated STAT3 activation.

To determine if TrkB was functionally crucial for the activation of STAT3 by DJ-1, we tested whether the activation of STAT3 in the presence of DJ-1 might depend on the expression of TrkB. The knockdown of TrkB significantly decreased the activation of STAT3, but the introduction of DJ-1 restores the level of phosphorylated STAT3. When we detected the endogenous DJ-1 expression in SNU387 TrkB-shRNA cells, we found the level of DJ-1 protein markedly reduced relative to its parental cells (Figs. 3e and S5A). Moreover, DJ-1 expression was significantly upregulated in PLC/PRF/5-TrkB and SNU387 cells compared to PLC/PRF/5 and SNU387 TrkB-shRNA cells, in contrast to corresponding transcription levels, which did not reveal significant differences by overexpression and knockdown of TrkB (Figs. 3f and S5B). Similar to the result of immunoblotting analysis, DJ-1 was localized in both the nuclei and cytoplasm of SNU387 cells and markedly increased relative to SNU387 TrkB-shRNA cells by immunostaining analyses (Fig. 3g).

To address this hypothesis, we determined whether TrkB interacts with DJ-1 directly. Interestingly, TrkB directly interacts with DJ-1 (Figs. 3h and S6A). Therefore, we next examined whether the kinase activity of TrkB is required for STAT3 activation by forming TrkB/DJ-1 complex. Interestingly, TrkB strongly interacted with DJ-1 relative to that of the kinase-dead mutant of TrkB (Figure S6A). Also, the introduction of TrkB into SNU387 TrkB-shRNA cells significantly induced the phospho-STAT3 expression compared to the kinase-dead mutant of TrkB (Figure S6B), indicating that the kinase activity of TrkB is essential for induction of STAT3 activation through the formation of the TrkB/DJ-1 complex. Therefore, we next examined whether TrkB induces DJ-1 stabilization. Indeed, TrkB leads to induce stabilization of DJ-1 via blocking degradation of ubiquitin-induced DJ-1 protein (Fig. 3i). Furthermore, the treatment of MG132, a proteasome inhibitor, in the presence of TrkB inhibits proteasome-mediated degradation of DJ-1 protein (Figure S7A, S7B, and S7C), suggesting that TrkB might regulate STAT3 activation via increasing stabilization of DJ-1 expression by inhibiting ubiquitin-mediated DJ-1 degradation. Thus, our findings emphasize that the upregulation of TrkB in HCC promotes tumorigenicity and metastasis of HCC via activation of STAT3 through induction of DJ-1 stabilization.

### Effects of TrkB-mediated induction of DJ-1 stability on the acquisition of cancer stem cells and chemoresistance.

**Fig. 4:**
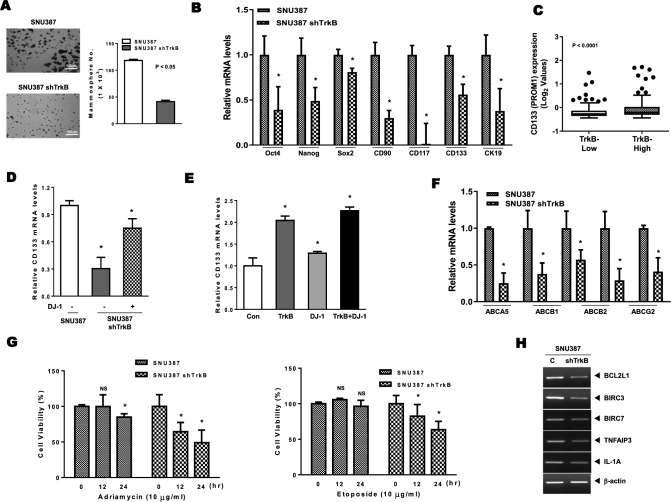
TrkB-mediated inhibition of DJ-1 degradation promotes the acquisition of cancer stem cell traits of hepatocellular carcinoma cells. **a** The quantification and images of the spheroid formation of SNU387 control- and TrkB-shRNA cells. The data are presented as three independent experiments’ mean ± SEM values. *P* < 0.05 (*), *t* test. **b** Relative mRNA expression of human embryonic stem (hES) cells markers (Oct4, Nanog, Sox2) and specific CSC markers (CD90, CD117, CD133, and CK19) of HCC in SNU387 control-shRNA or SNU387 TrkB-shRNA cells. The data are presented as three independent experiments’ mean ± SEM values. *P* < 0.05 (*), *t* test. **c** Relative mRNA expression of CD133 expression in human HCC patients between high or low TrkB expression. The average expression value of TrkB from the TCGA datasets was determined, rank ordered, and then divided into two equal groups. *P* < 0.0001, *t* test. **d** Relative mRNA expression of CD133 in SNU387 TrkB-shRNA cells transfected with DJ-1. The data are presented as three independent experiments’ mean ± SEM values. *P* < 0.005 (*), *t* test. **e** Relative mRNA expression of CD133 in PLC/PRF/5 cells transfected with DJ-1 or TrkB. The data are presented as three independent experiments’ mean ± SEM values. *P* < 0.005 (*), *t* test. **f** Relative mRNA expression of human ABC transporters (ABCA5, ABCB1, ABCB2, and ABCG2) in SNU387 control-shRNA or SNU387 TrkB-shRNA cells. The data are presented as three independent experiments’ mean ± SEM values. *P* < 0.05 (*), *t* test. **g** MTT analysis for cell survival in SNU387 control-shRNA or SNU387 TrkB-shRNA cells with treatment of Adriamycin and etoposide. The data are presented as three independent experiments’ mean ± SEM values. *P* < 0.05 (*); NS: not significant; *t* test. **h** RT-PCR analysis of mRNAs encoding anti-apoptotic markers (BCL2L1, BIRC3, BIRC7, TNFAIP3, and IL-1A) in SNU387 control-shRNA or SNU387 TrkB-shRNA cells. β-actin used as a loading control

In light of the upregulation of DJ-1, which has associated with liver stem/progenitor cell (LPCs) expansion [30] and self-renewal of glioblastoma cells [31], we asked whether TrkB-mediated inhibition of DJ-1 degradation was functionally crucial to acquiring traits of cancer stem cells (CSCs). To address this question, we determined whether TrkB promotes spheroid-forming ability associated with the presence of the human mammary stem/epithelial progenitor population [25]. Relative to the SNU387 control-shRNA cells, knockdown of TrkB in SNU387 cells markedly leads to loss of spheroid-forming ability (Fig. 4a), but upregulation of TrkB induces proliferation of spheroid-forming cells compared to parental PLC/PRF/5 cells (Figure S8). Therefore, we initially examined the expression of transcription factors (NANOG, OCT4, and SOX2), which acquire self-renewal traits and pluripotency in human embryonic stem (hES) cells [32]. The qRT-PCR analysis demonstrated that the loss of TrkB significantly decreases the accumulation of the CSC population by induction of CSC markers expression, including Nanog and SOX2, which are positively correlated with tumor grade and stage in bladder cancer [33] (Fig. 4b). We also confirmed the expression levels of specific surface markers of CSC of HCC, such as CD133 [34], CD90 [35], CD117 [36], and CK19 [37], which is associated with recurrence, the transition of CSC, and poor survival of HCC. Interestingly, TrkB induces the expression of mRNAs encoding these CSC markers in PLC/PRF/5-TrkB cells compared to PLC/PRF/5 cells (Figure S9A). We complemented the above results with luciferase activity. The knockdown of TrkB in SNU387 cells markedly suppresses the luciferase activities, which analyzes the activation of CD133 promoter, but the ectopic expression of TrkB significantly increases the luciferase activity of CD133 promoter (Figure S9B). We further confirmed that the gain of TrkB expression is markedly associated with the upregulation of CSC markers of HCC. A high level of TrkB showed upregulation of CD117, CK19, CD133, and CD90 expression from the TCGA dataset [21] relative to low TrkB expression (Figs. 4C and S9C), and its expression is significantly associated with CD117, CK19, CD133, and CD90 expression (Figs. 4d and S9D). Also, the introduction of DJ-1 in TrkB knockdown cells recovered the expression of CSC markers (Figure S10A), and upregulation of both TrkB and DJ-1 markedly increases the levels of CSC markers relative to induction of TrkB or DJ-1 (Figure S10C), indicating that increasing CSC population via the inhibition of DJ-1 degradation by TrkB enhances the aggressiveness of HCC and maintenance of mesenchymal/SC state [25]. In addition, STAT3 activation induces tumor formation and poor prognosis of HCC via upregulation of the CD133 expression by directly interacting with the CD133 promoter [38]. These previous studies suggest that activation of STAT3 by TrkB-mediated DJ-1 upregulation might force the transition of CSCs of HCC through induction of CD133 expression. To address this possibility, we analyzed the levels of CD133 expression in SNU387 TrkB-shRNA cells with or without DJ-1. CD133 expression by TrkB knockdown markedly decreased relative to the corresponding SNU387 cells, and these levels were rescued by ectopic DJ-1 expression (Figs. 4d and S10B). We further confirmed that both TrkB and DJ-1 significantly induce CD133 expression using qRT-PCR and luciferase reporter analysis (Figs. 4e and S10D). Once again, these observations demonstrated that TrkB-induced DJ-1 stabilization requires self-renewal traits to acquire high-grade malignancy.

The self-renewal capacity of CSCs contributes to the recurrence, metastasis, and multidrug/radiation resistance of cancer by inducing the expression of anti-apoptotic proteins and ATP-binding cassette (ABC) transporters [39]. We, therefore, asked whether the upregulation of TrkB confers therapeutic resistance through the upregulation of ABC transporters against the anticancer drug. The expression levels of ABCA5, ABCB1, ABCB2, ABCC1, and ABCG2 in SNU387 control-shRNA cells drastically increased relative to its TrkB-shRNA cells (Fig. 4f), and we found a similar expression pattern of mRNAs encoding these proteins observed in PLC/PRF/5-TrkB cells compared to its parental cells (Figure S11A), whereas the levels of ABCA1 and ABCA2 were not significantly different between both PLC/PRF/5 and PLC/PRF/5-TrkB cells or SNU387 control-shRNA and SNU387 TrkB-shRNA cells (data not shown). Moreover, both TrkB and DJ-1 markedly induced the levels of these mRNA expressions relative to TrkB or DJ-1 (Figure S11B), suggesting that increased DJ-1 stability by upregulation of TrkB acquires resistance to chemotherapeutic agents through induction of ABC transporters. We also assessed the effect of TrkB on chemoresistance. Interestingly, relative to SNU387 cells, either Adriamycin or etoposide treatments to SNU387 TrkB-shRNA cells inhibited cell viability time dependent (Fig. 4g). To address whether inhibition of cell death by TrkB was also instrumental in acquiring chemoresistance, we examined the expression of the anti-apoptotic markers to assess the effect of TrkB on apoptosis. The level of mRNAs encoding the anti-apoptotic markers (BCL2L1, BIRC3, BIRC7, TNFAIP3, and IL-1A) significantly upregulated in SNU387 control-shRNA cells relative to TrkB-shRNA cells (Fig. 4h). Our observations provided further evidence that upregulation of TrkB in HCC is necessary for eliminating conventional chemotherapy.

### Upregulation of STAT3 activation via TrkB-mediated inhibition of DJ-1 degradation is required for activation of the EMT program

**Fig. 5:**
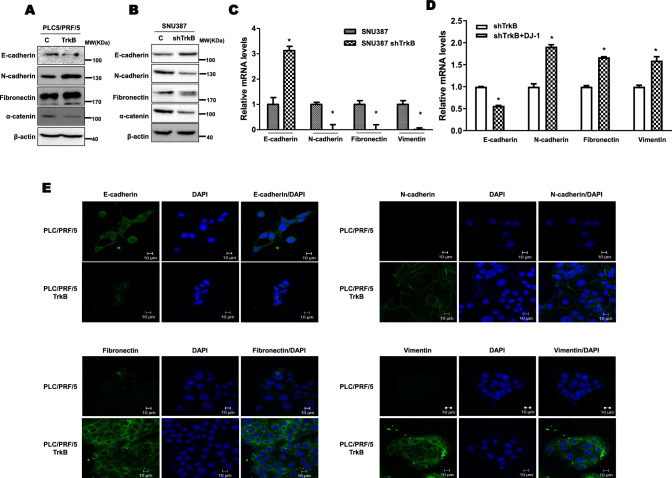
TrkB-mediated inhibition of DJ-1 strongly induces the activation of the EMT program of hepatocellular carcinoma cells. **a** Immunoblot analysis of fibronectin, α-catenin, E- and N-cadherin in PLC/PRF/5 or PLC/PRF/5 TrkB cells. β-actin used as a loading control. **b** Immunoblot analysis of fibronectin, TrkB, E- and N-cadherin in SNU387 control-shRNA or SNU387 TrkB-shRNA cells. β-actin used as a loading control. **c** Relative mRNA expression encoding fibronectin, vimentin, E- and N-cadherin in SNU387 control-shRNA or SNU387 TrkB-shRNA cells. The data are presented as three independent experiments’ mean ± SEM values. *P* < 0.05 (*), *t* test. **d** Relative mRNA expression of epithelial and mesenchymal markers in SNU387 TrkB-shRNA cells transfected with DJ-1. The data are presented as three independent experiments’ mean ± SEM values. *P* < 0.05 (*), *t* test. **e** Immunofluorescence images of expression of mesenchymal (fibronectin, vimentin, and N-cadherin) and epithelial (E-cadherin) markers in PLC/PRF/5 or PLC/PRF/5 TrkB cells. Scale bar = 10 µm

Recent reports have indicated that the EMT induces the acquisition of stemness via the transition from epithelial cell to CSC, which is capable of seeding new tumors by elevated tumor-initiating state [40–42]. Also, activation of the EMT program markedly elevated resistance to chemotherapeutic agents by increasing anti-apoptotic proteins and ABC transporter, leading to drug resistance of HCC [43–45]. However, it remained possible that TrkB-mediated inhibition of DJ-1 degradation may activate the transition into highly aggressive HCC cells via induction of an epithelial–mesenchymal transition (EMT) program, which is associated with the acquisition of stem cell-like properties, invasive and metastatic capacity, anticancer drug resistance, and tumor relapse [41, 42]. To investigate the functional role of TrkB in the induction of the EMT program in HCC, we initially focused on the expression change of epithelial and mesenchymal markers in PLC5/PRF/5-TrkB and SNU387 TrkB-shRNA cells. In immunostaining analysis, PLC5/PRF/5-TrkB cells markedly decreased protein levels of E-cadherin as an epithelial marker but significantly increased the levels of mesenchymal markers (N-cadherin, fibronectin, and vimentin) relative to PLC5/PRF/5 cells (Fig. 5a). However, in contrast, the knockdown of TrkB in SNU387 cells exhibited opposite results from results shown in PLC5/PRF/5-TrkB cells. The loss of TrkB expression in SNU387 cells leads to reduced mesenchymal markers and induction of E-cadherin relative to SNU387 control-shRNA cells (Fig. 5b). A similar expression pattern of mRNAs encoding epithelial and mesenchymal markers was observed in PLC5/PRF/5-TrkB and SNU387 control-shRNA cells relative to PLC5/PRF/5 and SNU387 TrkB-shRNA cells (Figs. 5c and S12A). Also, introducing DJ-1 in TrkB knockdown cells showed similar patterns of epithelial and mesenchymal markers in SNU387 control cells (Fig. 5d). As gauged by immunofluorescence and real-time RT-PCR (qRT-PCR) analysis, immunostaining of E-cadherin revealed downregulation significantly, but N-cadherin, fibronectin, and vimentin expression levels markedly increased in PLC5/PRF/5-TrkB cells relative to PLC5/PRF/5 cells (Figs. 5e and Figures S12A). Also, both DJ-1 and TrkB significantly induce the mesenchymal markers and reduce the epithelial markers more than when TrkB is present alone. (Figure S12B).

**Fig. 6:**
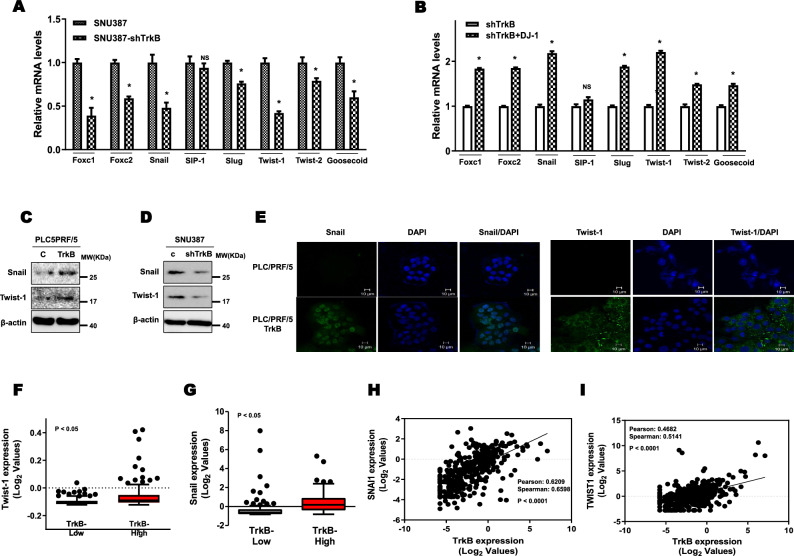
Upregulation of EMT-TFs by inducing TrkB-mediated inhibition of DJ-1 degradation promotes the induction of the EMT in hepatocellular carcinoma cells. **a** Relative mRNA expression of EMT-TFs including Foxc1, Foxc2, Snail, SIP1, Slug, Twist-1, Twist-2, and Goosecoid in SNU387 control-shRNA or SNU387 TrkB-shRNA cells. The data are presented as three independent experiments’ mean ± SEM values. *P* < 0.05 (*); NS: not significant; *t* test. **b** Relative mRNA expression of EMT-TFs in SNU387 TrkB-shRNA cells transfected with DJ-1. The data are presented as three independent experiments’ mean ± SEM values. *P* < 0.05. **c** Immunoblot analysis of Snail and Twist-1 in PLC/PRF/5 or PLC/PRF/5 TrkB cells. β-actin used as a loading control. **d** Immunoblot analysis of Snail and Twist-1 in SNU387 control-shRNA or SNU387 TrkB-shRNA cells. β-actin used as a loading control. **e** Immunofluorescence images of expression of Snail and Twist-1 in PLC/PRF/5 or PLC/PRF/5 TrkB cells. Scale bar = 10 µm. **f** Box-and-whisker (Tukey) plots of Twist-1 expression in human HCC patients between high or low TrkB expression. The average expression value of TrkB from the TCGA datasets was determined, rank ordered, and then divided into two equal groups (*N* = 372). *P* < 0.05, *t* test. **g** Box-and-whisker (Tukey) plots of Snail expression in human HCC patients between high or low TrkB expression. The average expression value of TrkB from the TCGA datasets was determined, rank ordered, and then divided into two equal groups (*N* = 372). *P* < 0.05, *t* test. **h** The correlation of TrkB and Snail expression in TCGA datasets (*N* = 372). *P* < 0.0001; Spearman's correlation coefficient. **i** The correlation of TrkB and Twist-1 expression in TCGA datasets (*N* = 372). *P* < 0.0001; Spearman’s correlation coefficient

We next investigated the effects of TrkB-mediated inhibition of DJ-1 degradation on the regulation of EMT-inducing transcription factors (EMT-TFs), which play critical roles in the acquisition of critical malignant traits [46]. The qRT-PCR analysis revealed that TrkB overexpression in PLC5/PRF/5 cells significantly upregulated the expression of EMT-TFs, including Foxc1, Foxc2, Snail, Twist-1, Twist-2, and Goosecoid, but not in Slug and SIP1. In contrast, SNU387 TrkB-shRNA cells markedly reduced expression of EMT-TFs such as Foxc1, Foxc2, Snail, Twist-1, Twist-2, and Goosecoid, whereas the levels of SIP1 and Slug did not reveal significant differences from that of control cells (Figs. 6a and S15A). Also, the introduction of DJ-1 in SNU387 TrkB-shRNA cells showed a significant recovered reduction of EMT-TF expression (Fig. 6b). We then examined the expression of Snail and Twist-1 protein levels, which are the key inducer of EMT and CSC transition. As anticipated, PLC/PRF/5-TrkB cells upregulated Snail and Twist-1 expression, whereas these levels were markedly decreased in SNU387 TrkB-shRNA cells relative to control-shRNA (Fig. 6c, d). These observations were confirmed by comparing immunostaining analysis between PLC/PRF/5-TrkB and control cells. Immunostaining analysis of Snail and Twist-1, as well as immunoblotting and qRT-PCR analysis, exhibited that Snail and Twist-1 highly increased PLC/PRF/5-TrkB cells relative to control cells (Fig. 6e). Taken together, we provided further evidence that the gain of TrkB expression is markedly associated with EMT-TFs expression using public microarray data. A high level of TrkB increases the expression of EMT-TFs (FOXC1, FOXC2, Snail, ZEB1, ZEB2, Twist-1, and Twist-2) from the TCGA dataset [21] relative to low TrkB expression (Figs. 6f, g, and S13), and we also identified the direct correlation between TrkB and the level of EMT-TFs (FOXC1, FOXC2, GSC, Snail, ZEB1, ZEB2, Twist-1, and Twist-2) via correlation analysis using TCGA dataset (Figs. 6h, i, and S14). Moreover, both TrkB and DJ-1 significantly induce the EMT program by upregulating the expression of EMT-TFs (Figures S15A and S15B). These observations exhibited that TrkB-mediated inhibition of DJ-1 degradation in HCC might be linked to high tumorigenicity and acquisition of CSC traits via induction of EMT.

### Contribution of TrkB in primary tumor formation and metastasis in vivo

**Fig. 7:**
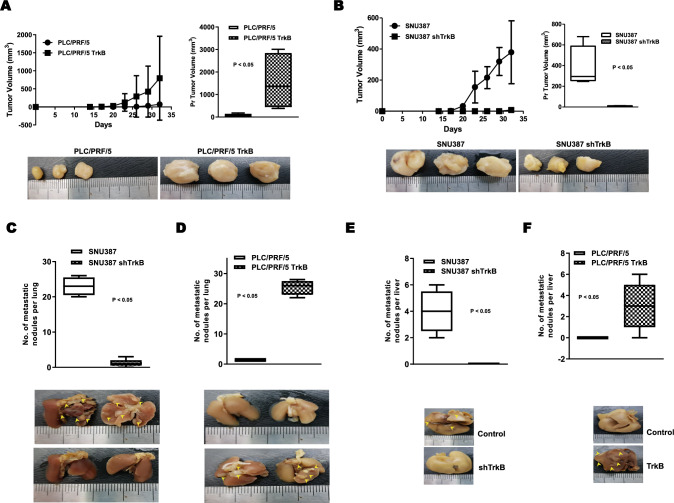
TrkB promotes tumorigenicity and metastasis of hepatocellular carcinoma cells in vivo. **a** In vivo tumor formation (upper) and the representative images (lower) of PLC/PRF/5 or PLC/PRF/5 TrkB cells in the mammary fat pads of mice. The error bar is presented as the mean ± standard error of the mean (SEM). The experiments were performed in triplicate. *P* < 0.05; *t* test. *n* = 7 mice for PLC/PRF/5 group; *n* = 7 mice for PLC/PRF/5 TrkB group. **b** In vivo tumor formation (upper) and the representative images (lower) of SNU387 control-shRNA or SNU387 TrkB-shRNA cells in the mammary glands of mice. The experiments were performed in triplicate. *P* < 0.05; *t* test. *n* = 7 mice for SNU387 group; *n* = 7 mice for SNU387 shTrkB group. **c** Quantification (upper) and representative images (lower) of lung surface metastatic foci of SNU387 control-shRNA or SNU387 TrkB-shRNA cells. The experiments were performed in triplicate. *P* < 0.05; *t* test. *n* = 7 mice for SNU387 group; *n* = 7 mice for SNU387 shTrkB group. **d** Quantification (upper) and representative images (lower) of lung surface metastatic foci of PLC/PRF/5 or PLC/PRF/5 TrkB cells. The experiments were performed in triplicate. *P* < 0.05; *t* test. *n* = 7 mice for PLC/PRF/5 group; *n* = 7 mice for PLC/PRF/5 TrkB group. **e** Quantification (upper) and representative images (lower) of liver surface metastatic foci of SNU387 control-shRNA or SNU387 TrkB-shRNA cells. *P* < 0.05; *t* test. *n* = 7 mice/group. **f** Quantification (upper) and representative images (lower) of liver surface metastatic foci of PLC/PRF/5 or PLC/PRF/5 TrkB cells. The experiments were performed in triplicate. *P* < 0.05; *t* test. *n* = 7 mice for PLC/PRF/5 group; *n* = 7 mice for PLC/PRF/5 TrkB group

The above observations revealed that upregulation of TrkB and TrkB-mediated inhibition of DJ-1 was important for enhancing the metastatic potential and survival of HCC cells. We, therefore, further addressed the possibility that induction of metastatic potential of HCC cells by upregulation of TrkB leads to primary tumor formation in vivo. Tumor formation from mice harboring PLC/PRF/5-TrkB cells significantly increased than those of mice harboring PLC/PRF/5 cells (Fig. 7a). Also, similar to the observed increased tumor formation of PLC/PRF/5-TrkB cells, injection with SNU387 control-shRNA cells formed large tumors in mice, whereas tumor incidence was markedly decreased upon implantation of SNU387 TrkB-shRNA cells (Fig. 7b).

We subsequently determined whether the expression of TrkB affect further the formation of metastatic foci in vivo. The number of metastatic foci in the lung induced 19.1-fold and 18.1-fold in PLC/PRF/5-TrkB and SNU387 control-shRNA cells than the mice bearing PLC/PRF/5 and SNU387 TrkB-shRNA cells, respectively (Fig. 7c, d). Furthermore, the upregulation of TrkB led to a fourfold and threefold induction in liver metastases in mice harboring PLC/PRF/5-TrkB and SNU387 control-shRNA cells relative to mice harboring PLC/PRF/5 and SNU387 TrkB-shRNA cells (Fig. 7e, f). These results suggested that TrkB expression is required for the formation and progression of HCC.

## Discussion

Although the expression of TrkB has been associated with the pathogenesis of various cancers, the function of TrkB has been partially identified in some of the previous studies. Also, TrkB-mediated acquirement of the ability to overcome multiple barriers to dissemination in HCC is still mostly unknown. Our observations presented here demonstrate that TrkB plays an essential role in activating cell properties associated with tumor-initiating, aggressiveness, and transition of cancer stem cell (CSC) states. We found that the upregulation of TrkB markedly increased in highly metastatic HCC cell lines and tumor samples of HCC patients. TrkB also strongly enhances various steps of metastatic cascades in HCC cells in vivo and in vitro. These observations suggested that TrkB may regulate mesenchymal traits to disseminate to various distant organs.

DJ-1 induces Bcl-X_L_ stabilization through the complex formation to protect cell death in Parkinson’s disease and cancer [47]. Also, DJ-1 is involved in acquiring resistance against chemotherapeutic drugs, including cisplatin, Adriamycin, and etoposide [48, 49]. However, the degradation of DJ-1 and the signaling mechanism that maintains the stability of DJ-1 in cancer have remained unclear. We find here that TrkB increases the stabilization of DJ-1 via direct interaction known as an enhancer of cancer cell survival, metastasis, and chemoresistance. Also, the ectopic expression of TrkB significantly induces phosphorylated STAT3 levels and enhances STAT3 activation by induction of DJ-1-STAT3 complex formation.

The activation of EMT by the EMT-TFs is known to confer multiple traits associated with high-grade malignancy to tumor-initiating carcinoma cells and establish the progression of the malignant tumor. EMT-TFs, including forkhead box C proteins Foxc1 and Foxc2, Snail, Slug, Sip1, Goosecoid, and the basic helix-loop-helix factors Twist-1 and Twist-2, frequently regulate the expression of one another and, in various combinations, lead to induction of oncoproteins and repression of tumor suppressors to maintain mesenchymal traits [25, 40–42]. Although emerging functions of the EMT program have been investigated in HCC, there is still little known that the molecular mechanism underlying EMT and genes inducing more mesenchymal and invasive states contribute functionally to tumor metastasis and chemoresistance. We observed that TrkB-mediated upregulation of DJ-1 stability activates tumor progression to malignancy by inducing an EMT program via upregulation of EMT-TFs, including Foxc1 and Foxc2, Snail, Goosecoid, Twist-1, and Twist-2. Taken together, we showed that increasing DJ-1 expression by TrkB induces and maintains stem cell states of HCC by upregulation of stem cell markers of HCC such as Sox2, Nanog, and Oct4. Our findings, in concordance with accumulating evidence, suggested that the activation of EMT via inhibition of DJ-1 degradation by TrkB induces entrance to the CSC state by acquiring cellular traits to arising CSC-enriched subpopulation [46, 50].

Once the EMT program drives cancer cells into the CSC state, the CSC subpopulation in cancer cells leads to clinical relapse via acquiring drug resistance by increasing the level of ABC transporters and CSC markers [43, 44, 46, 51, 52]. Thus, our observations revealed that upregulation of TrkB and TrkB-mediated inhibition of DJ-1 degradation increases spheroid formation, associated with mammary progenitor/stem cells by inducing ABC transporters (ABCA5, ABCB1, ABCB2, ABCC1, and ABCG2) and CSC markers (CD133, CD90, CD117, CK19, Oct3, and Nanog). In addition, we have also demonstrated that the induction of anti-apoptotic proteins by upregulation of TrkB leads to resistance to chemotherapy via inhibiting cell death by Adriamycin (doxorubicin) and etoposide, which are known chemotherapy medicines. These observations may provide that the physiologic relevance of TrkB in the progression of HCC critically contributes to inducing and maintaining the generation of unlimited numbers of cancer stem cells and chemoresistance. Therefore, TrkB kinase activity disruption may efficiently eliminate the resistance of conventional chemotherapy or radiotherapy, and the functional roles of TrkB in maintaining CSC state and therapeutic interruption will be addressed in further study.

### Supplementary Information

Below is the link to the electronic supplementary material.

Supplementary file1 (DOCX 24 KB)

Supplementary file2 (PDF 1255 KB)

Supplementary file3 (DOCX 21 KB)

## Data Availability

The datasets used and/or analyzed during the current study are available from the corresponding author upon reasonable request.
