# Supplementary material for: TrkB inhibition of DJ-1 degradation promotes the growth and maintenance of cancer stem cell characteristics in hepatocellular carcinoma
Source: Cell Mol Life Sci. 2023 Sep 25;80(10):303. doi: 10.1007/s00018-023-04960-z (PMC10520132; doi:10.1007/s00018-023-04960-z)
Supplement: Supplementary file 1 — Supplementary file1 (DOCX 24 KB) [file 18_2023_4960_MOESM1_ESM.docx]

**Table S1. Primer sequences for PCR and real-time RT-PCR**

| **Real-time RT-PCR or RT-PCR Primers** | |
| --- | --- |
| **Gene** | **Primers** |
| **Human CD133** | F: 5’- CCTGGGGCTGCTGTTTATTA -3’  R: 5’- TCACCAACAGGGAGATTGCAAA -3’ |
| **Human CD117** | F: 5′- AGAGACTTGGCAGCCAGAAA -3′  R: 5′- TGCCATCCACTTCACAGGTA -3′ |
| **Human CD90** | F: 5’- TCCAGGCCACGGATTTCAT -3’  R: 5’- CCCACTTCTCCTCAAGGTTTGA -3’ |
| **Human CK19** | F: 5’- TGAGCAGGTCCGAGGTTACT -3’  R: 5’- TCTTCCAAGGCAGCTTTCAT -3’ |
| **Human Oct4** | F: 5’- ACATCAAAGCTCTGCAGAAAGAACT -3’  R: 5’- CTGAATACCTTCCCAAATAGAACCC -3’ |
| **Human Nanog** | F: 5’- CAGCTGTGTGTACTCAATGATAGATTT -3’  R: 5’- ACACCATTGCTATTCTTCGGCCAGTTG -3’ |
| **Human SOX2** | F: 5’- AAATGGGAGGGGTGCAAAAGAGGAG -3’  R: 5’- CAGCTGTCATTTGCTGTGGGTGATG -3’ |
| **Human ABCA1** | F: 5’- AACAGTTTGTGGCCCTTTTG -3’  R: 5’- AGTTCCAGGCTGGGGTACTT -3’ |
| **Human ABCA2** | F: 5’- AGCTGCTGCTCTGGAAGAAC -3’  R: 5’- GCCGCTGTGTAGAAGGAGAC -3’ |
| **Human ABCA5** | F: 5’- CCACTGGAGGAAGAAATGGA -3’  R: 5’- AAAGGCTCATGGTGCTCACT -3’ |
| **Human ABCB1** | F: 5’- CTATGCTGGATGTTTCCGGT -3’  R: 5’- GCTTTGGCATAGTCAGGAGC -3’ |
| **Human ABCB2** | F: 5’- ACGTCCACCCTGAGTGATTC -3’  R: 5’- GACACTGATCCCAGAGCAT -3’ |
| **Human ABCC1** | F: 5’- AAGAAAACAGGGAAGCAGCA -3’  R: 5’- GCTCTCTGGGTTTGAAGTCG -3’ |
| **Human ABCG2** | F: 5’- ATCTTGGCTGTCATGGCTTC -3’  R: 5’- TCTTCGCCAGTACATGTTGC -3’ |
| **Human FOXC1** | F: 5’- ACGGCATCTACCAGTTCATC -3’  R: 5’- TCCTTCTCCTCCTTGTCCTT -3’ |
| **Human FOXC2** | F: 5’- GCCTAAGGACCTGGTGAAGC -3’  R: 5’- TTGACGAAGCACTCGTTGAG -3’ |
| **Human E-cadherin** | F: 5’- TGCCCAGAAAATGAAAAAGG -3’  R: 5’- GTGTATGTGGCAATGCGTTC -3’ |
| **Human N-cadherin** | F: 5’- ACAGTGGCCACCTACAAAGG -3’  R: 5’- CCGAGATGGGGTTGATAATG -3’ |
| **Human Fibronectin** | F: 5’- CAGTGGGAGACCTCGAGAAG -3’  R: 5’- TCCCTCGGAACATCAGAAAC -3’ |
| **Human Vimentin** | F: 5’- GAGAACTTTGCCGTTGAAGC -3’  R: 5’- GCTTCCTGTAGGTGGCAATC -3’ |
| **Human Snail** | F: 5’- CCTCCCTGTCAGATGAGGAC -3’  R: 5’- CCAGGCTGAGGTATTCCTTG -3’ |
| **Human SIP1** | F: 5’- TTCCTGGGCTACGACCATAC -3’  R: 5’- TGTGCTCCATCAAGCAATTC -3’ |
| **Human Slug** | F: 5’- GGGGAGAAGCCTTTTTCTTG -3’  R: 5’- TCCTCATGTTTGTGCAGGAG -3’ |
| **Human Goosecoid** | F: 5’- GAAGGTAAAAGCGATTTGGA -3’  R: 5’- ACATCGCCATCACTTTATTG -3’ |
| **Human TrkB** | F: 5’- AACATTTCCGTCACCTTGACTTGT -3’  R: 5’- AATGTCACAGGAGCATGTAAATGG -3’ |
| **Cloning Primers** | |
| **Gene** | **Primers** |
| **TrkB** | F: 5’- ACCATGTCGCCCTGGCCGAGGTGGCAT -3’  R: 5’- GCCTAGGATGTCCAGGTAGACGGG-3’ |
| **TrkB-shRNA** | F: 5’-’ CCGGCTGACGCAGTCGCAGATGCCTCGAG GCATCTGCGACTGCGTCAGTTTTTG -3’  R: 5’- AATTCAAAAACTGACGCAGTCGCAGATGC CTCGAGGCATCTGCGACTGCGTCAG -3’ |

**Table S2. ANTIBODY RESOURCE TABLE**

| **FOR WESTERN BLOTTING** | | | |
| --- | --- | --- | --- |
| ANTIBODIES | SOURCE | IDENTIFIER(Cat.) | DILUTION |
| E-cadherin | Cell Signaling Technology | #3195S | 1:1000 |
| N-cadherin | Cell Signaling Technology | #26836S | 1:1000 |
| Fibronectin | Cell Signaling Technology | #13116S | 1:1000 |
| alpha-catenin | Cell Signaling Technology | #2131S | 1:1000 |
| Vimentin | Cell Signaling Technology | #5741S | 1:1000 |
| STAT3 | Cell Signaling Technology | #9139S | 1:1000 |
| Snail | Cell Signaling Technology | #3879S | 1:1000 |
| phospho-STAT3 | Cell Signaling Technology | #9145S | 1:1000 |
| TrkB | Abcam | Ab18987 | 1:1000 |
| Twist-1 | Abcam | Ab50887 | 1:1000 |
| DJ-1 | Abcam | Ab18257 | 1:1000 |
| HA | Santa Cruz Biotechnology | sc-7392 | 1:1000 |
| GFP | Santa Cruz Biotechnology | sc-9996 | 1:1000 |
| V5 | Life Technologies | MA5-15253 | 1:1000 |
| β-actin | Sigma | A1978 | 1:1000 |
| Flag | Sigma | F3165 | 1:1000 |
| Anti-mouse | Cell Signaling Technology | #7076 | 1:3000 |
| Anti-rabbit | Cell Signaling Technology | #7074 | 1:3000 |
| **FOR IMMUNOPRECIPITATION, IMMUNOFLUORESCENCE**  **AND IMMUNOHISTOCHEMISTRY** | | | |
| ANTIBODIES | SOURCE | IDENTIFIER(Cat.) | DILUTION |
| GFP | Santa Cruz Biotechnology | sc-9996 | 1:100 |
| Flag | Sigma | F3165 | 1:100 |
| V5 | Life Technologies | MA5-15253 | 1:100 |
| E-cadherin | Cell Signaling Technology | #3195S | 1:100 |
| N-cadherin | Cell Signaling Technology | #26836S | 1:100 |
| Fibronectin | Cell Signaling Technology | #13116S | 1:100 |
| Vimentin | Cell Signaling Technology | #5741S | 1:100 |
| Snail | Cell Signaling Technology | #3879S | 1:100 |
| DJ-1 | Abcam | Ab18257 | 1:100 |
| TrkB | Abcam | Ab18987 | 1:100 |
| Twist-1 | Abcam | Ab50887 | 1:100 |
| Anti-mouse | Cell Signaling Technology | #4408 | 1:3000 |
| Anti-mouse | Cell Signaling Technology | #8890 | 1:3000 |
| Anti-rabbit | Cell Signaling Technology | #4412 | 1:3000 |
| Anti-rabbit | Cell Signaling Technology | #8889 | 1:3000 |
