# Supplementary figures and images for: TrkB inhibition of DJ-1 degradation promotes the growth and maintenance of cancer stem cell characteristics in hepatocellular carcinoma
Source: Cell Mol Life Sci. 2023 Sep 25;80(10):303. doi: 10.1007/s00018-023-04960-z (PMC10520132; doi:10.1007/s00018-023-04960-z)

**pGL3-STAT3**

(Sequence ID : NM\_213662)

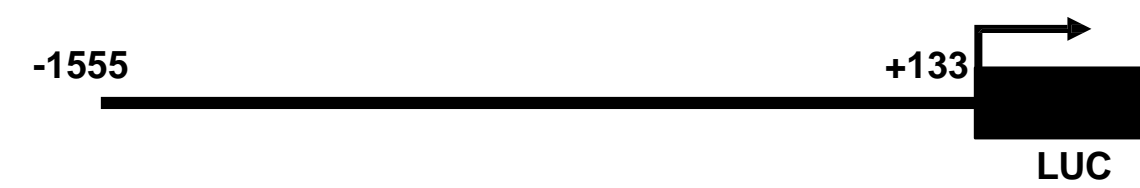**pGL3-CD133**

(Sequence ID : NM\_001145847)

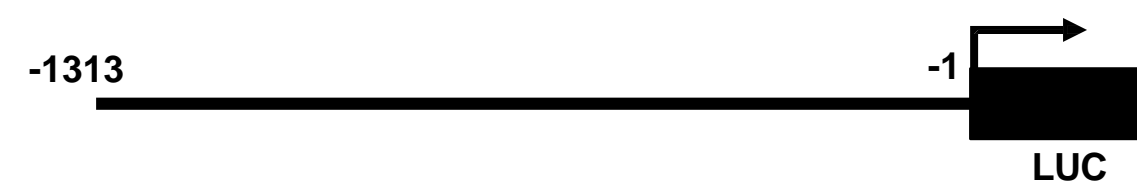

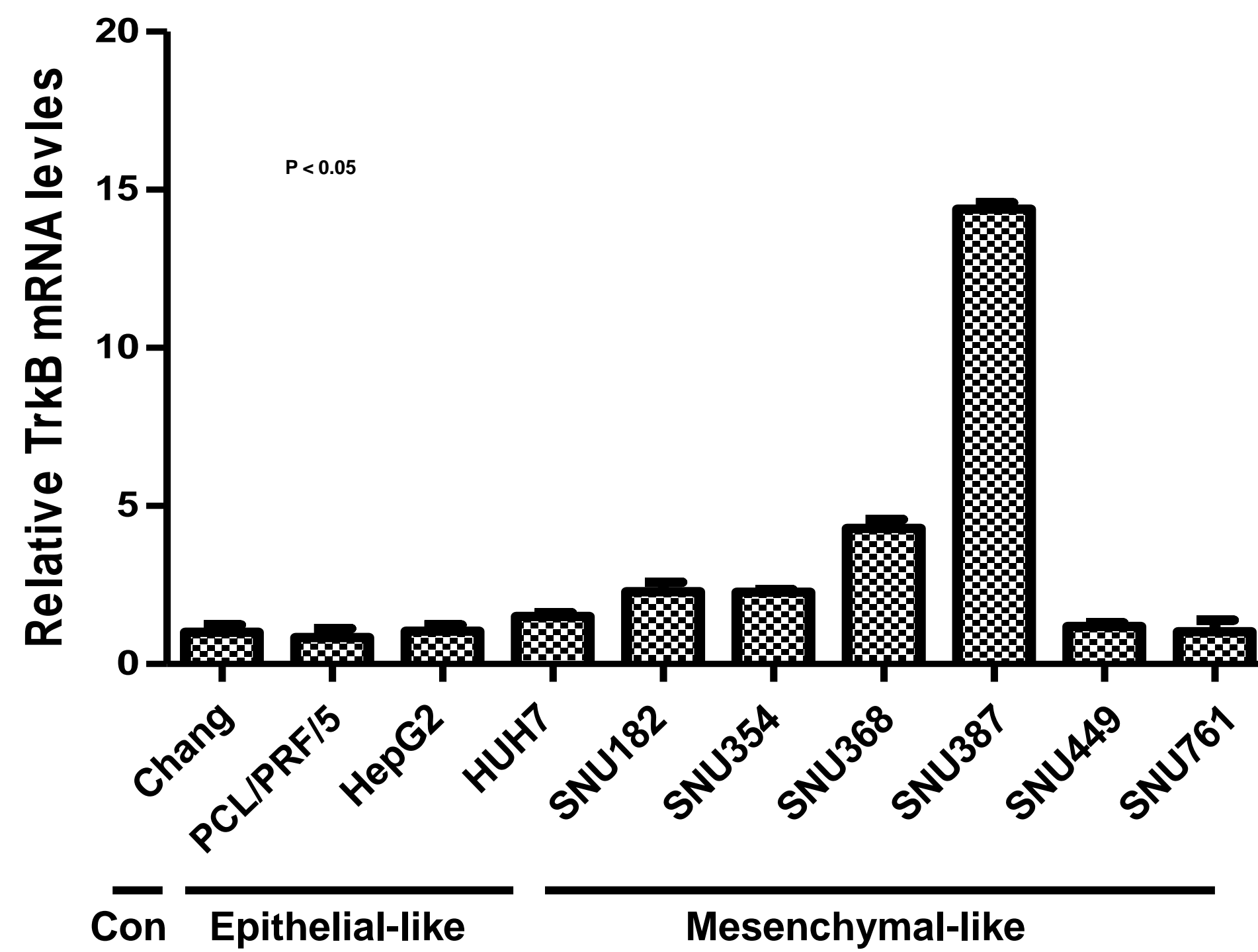

**A**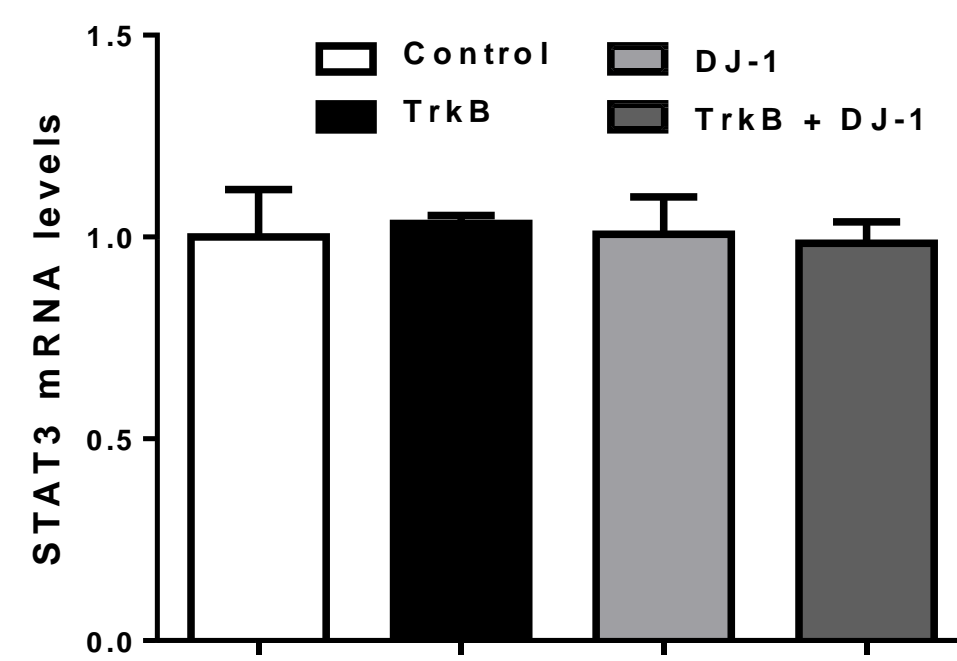**B**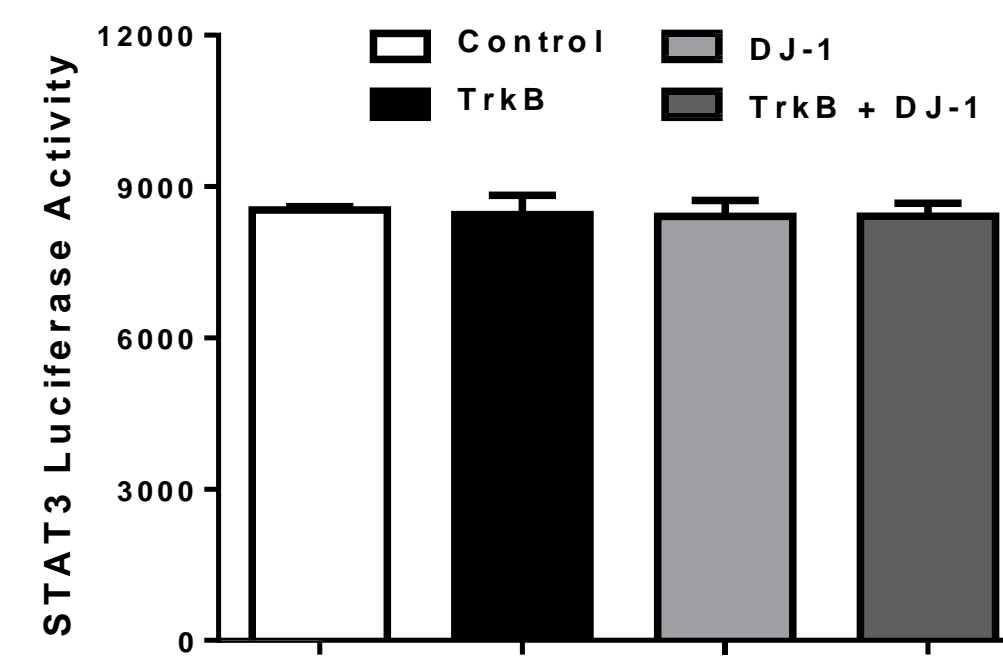

**A**

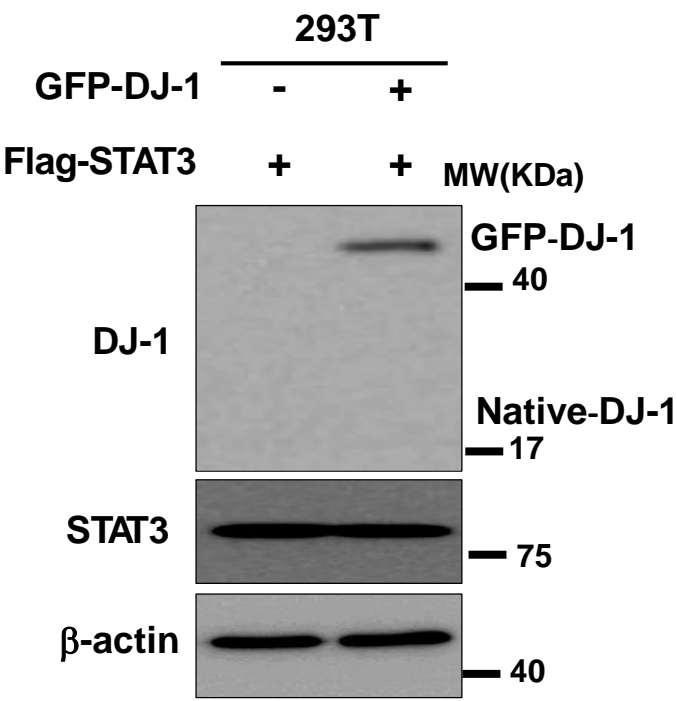

**B**

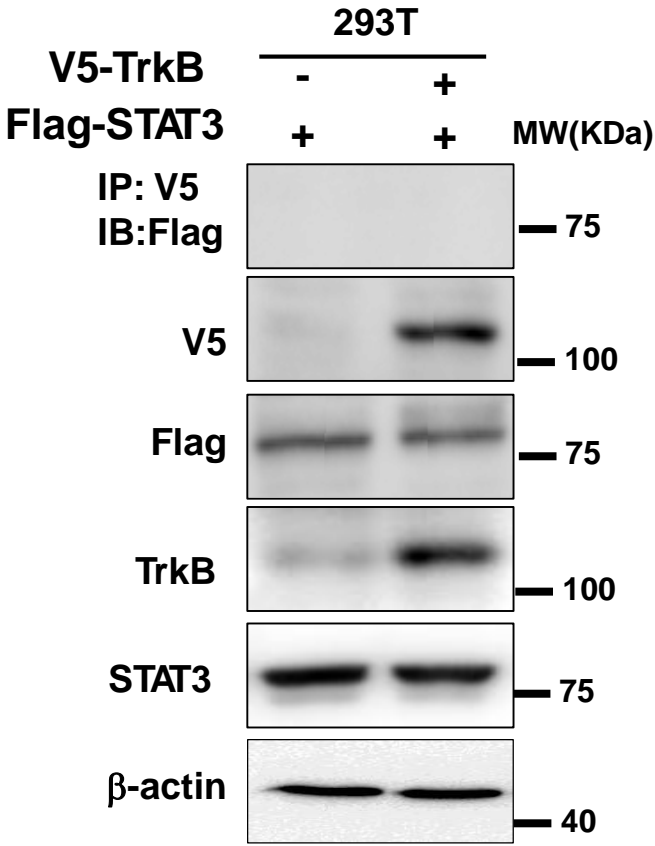

**C**

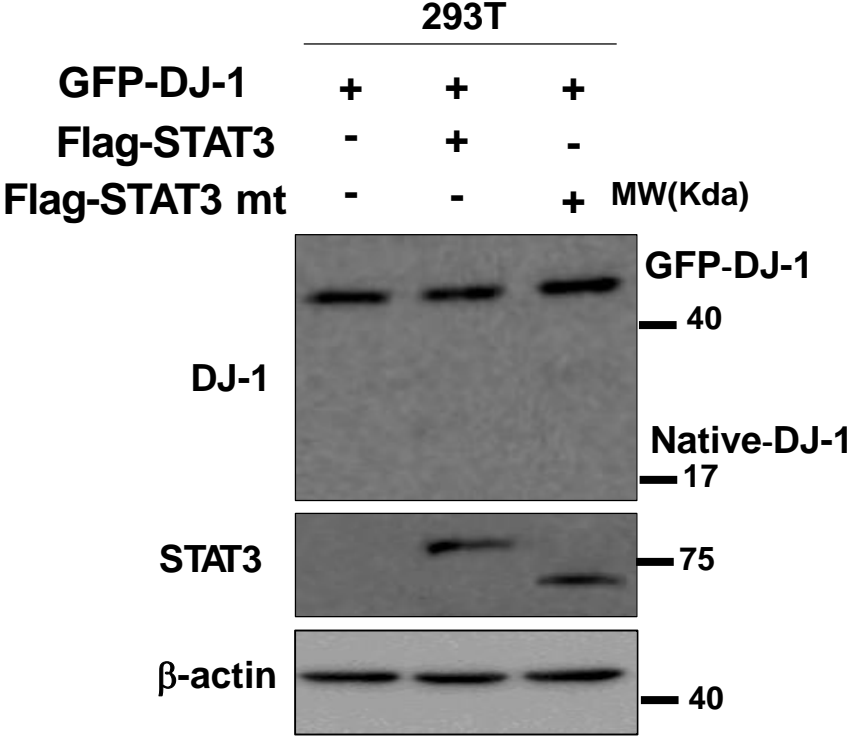

**A**

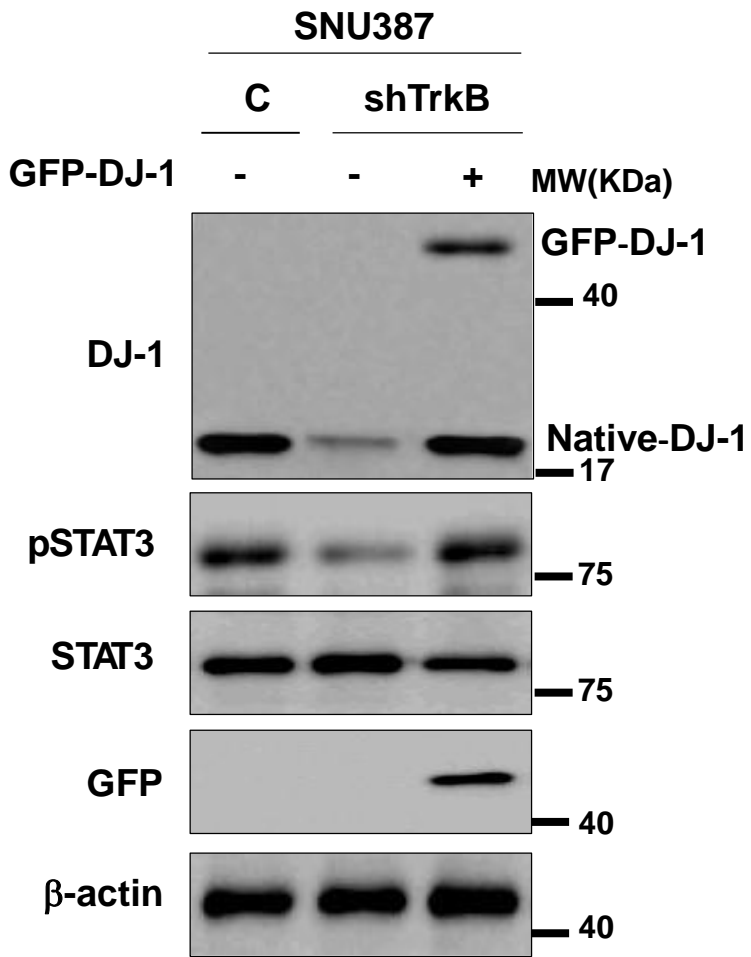

**B**

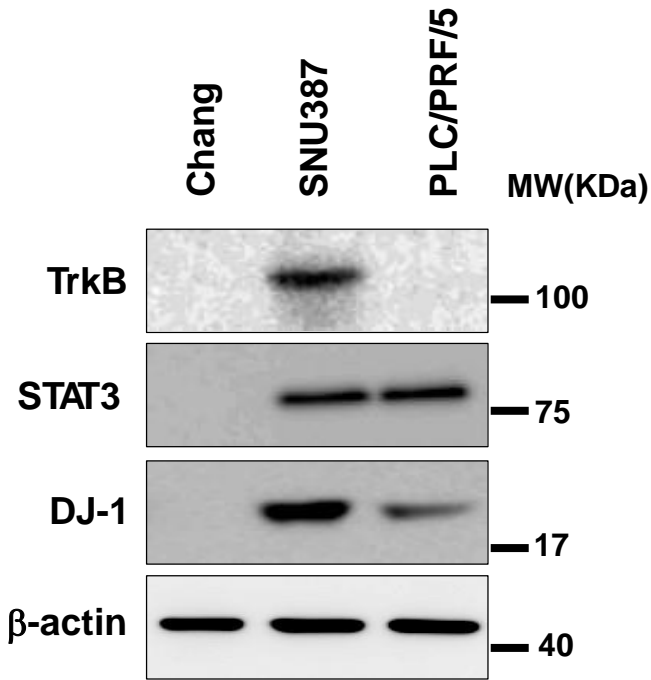

**A**

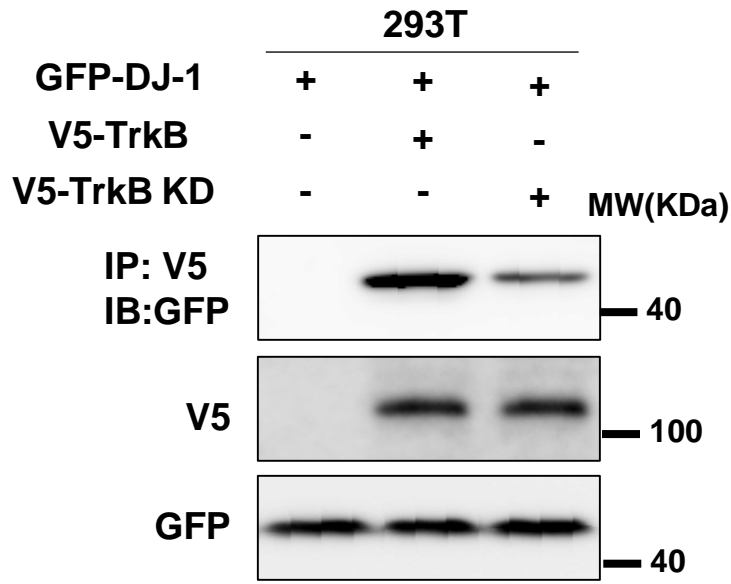

**B**

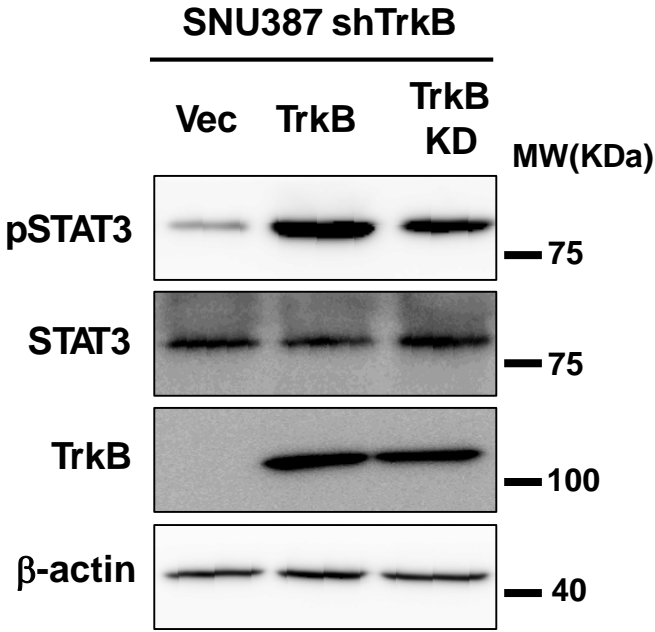

**A**

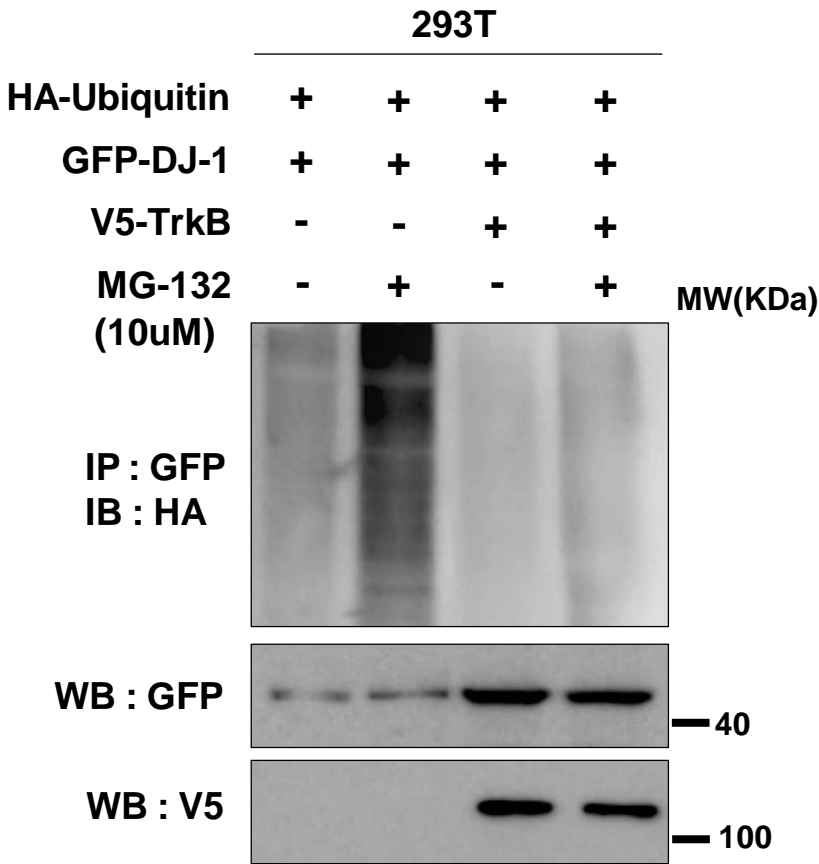

**B**

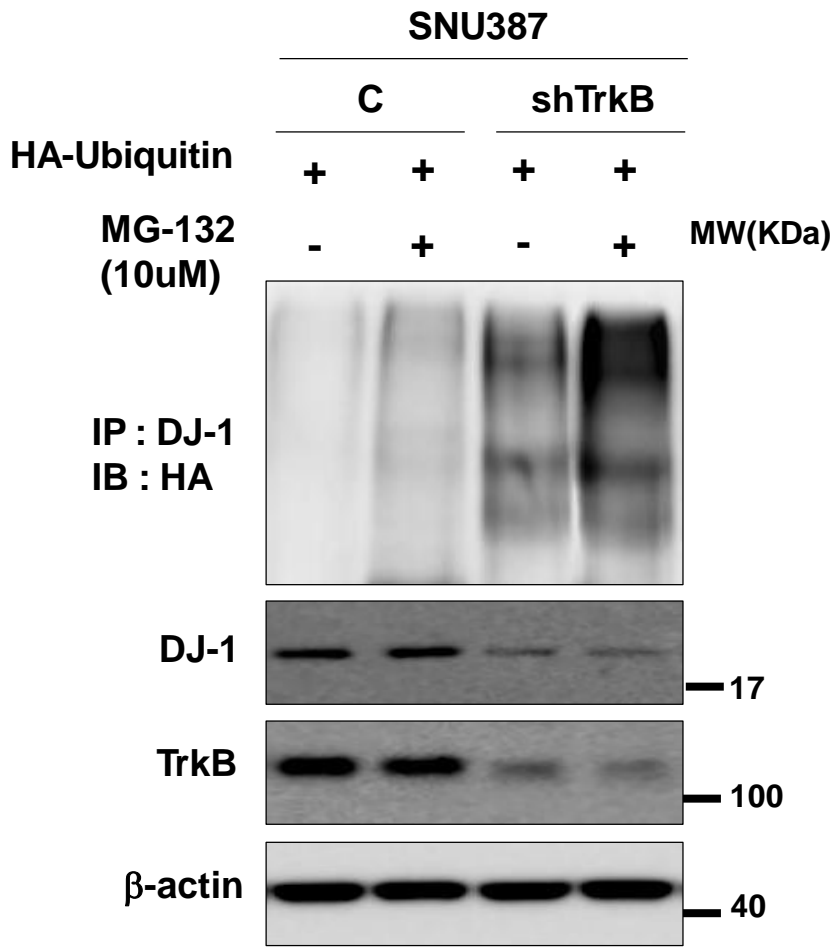

**C**

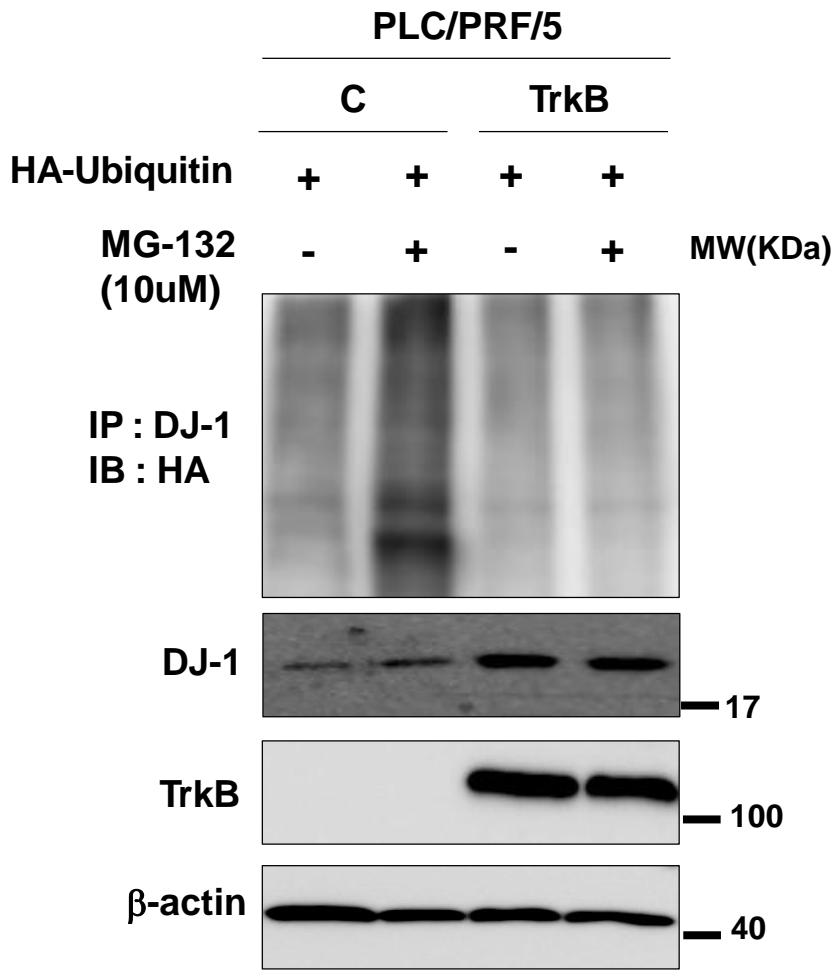

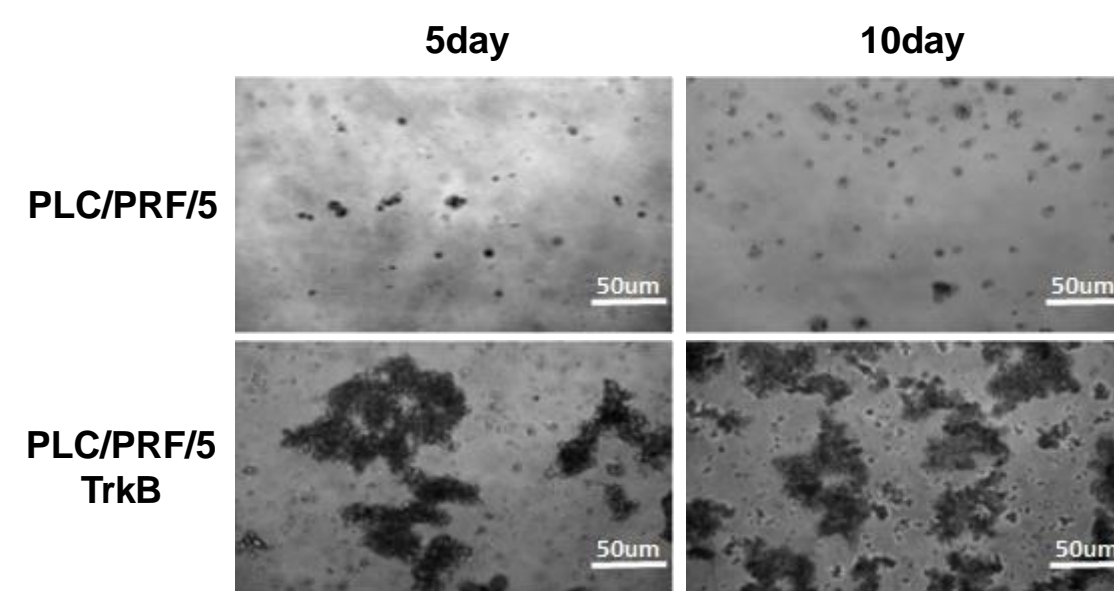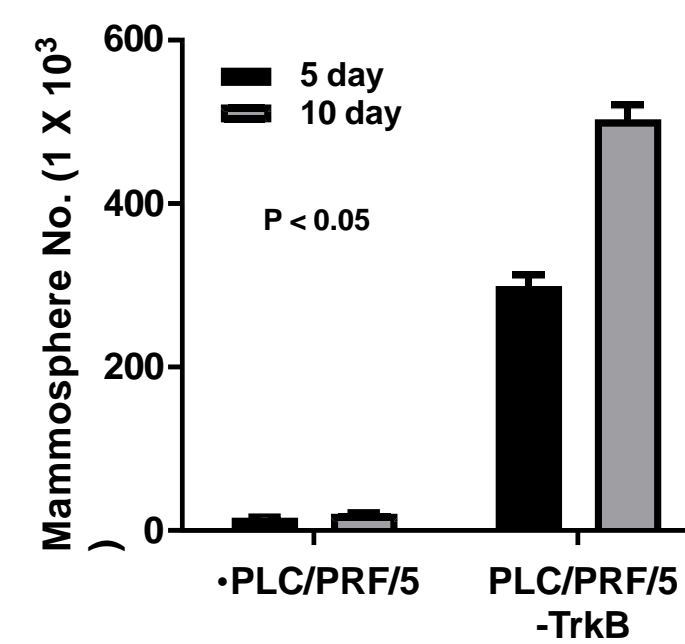

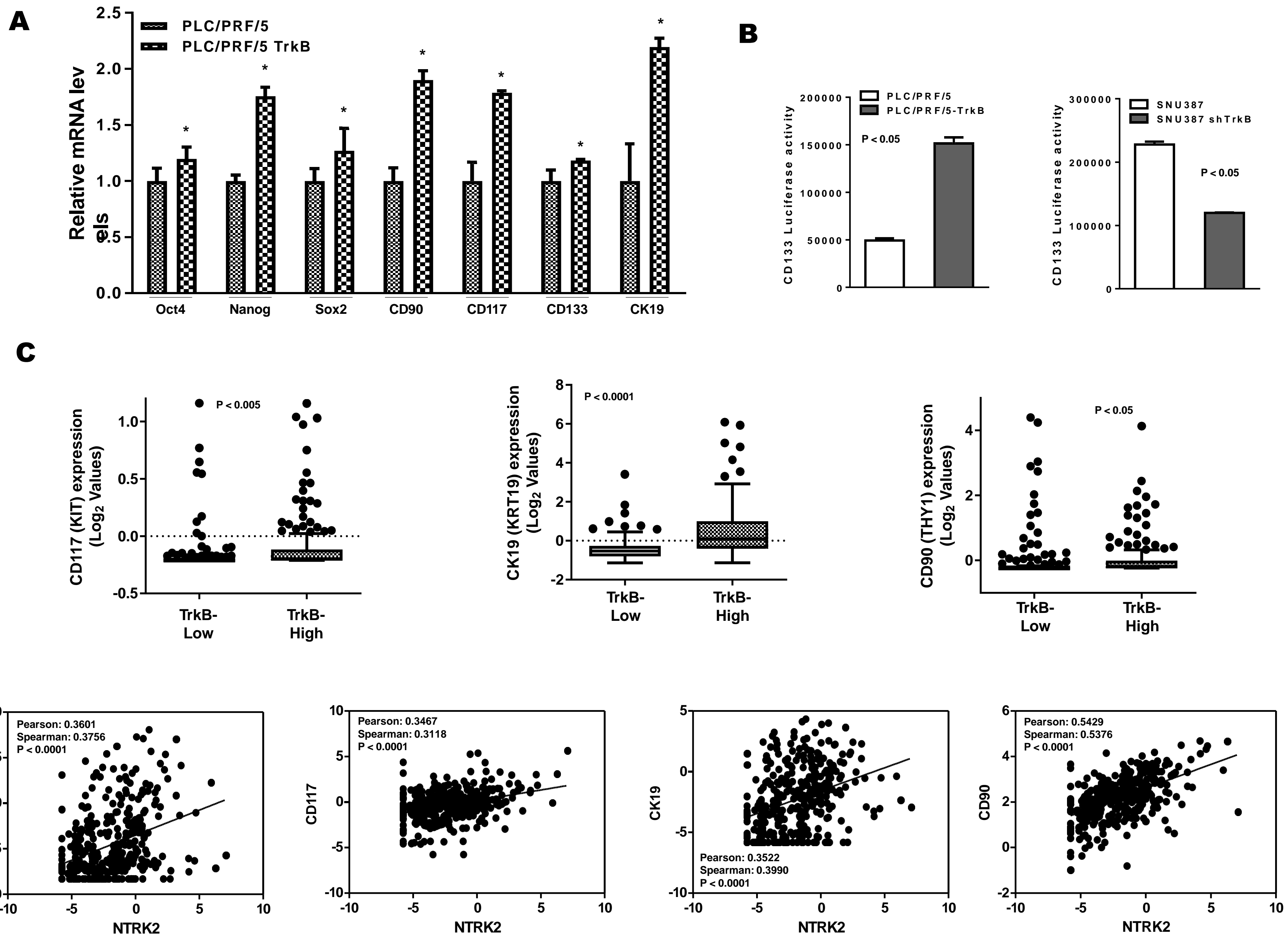

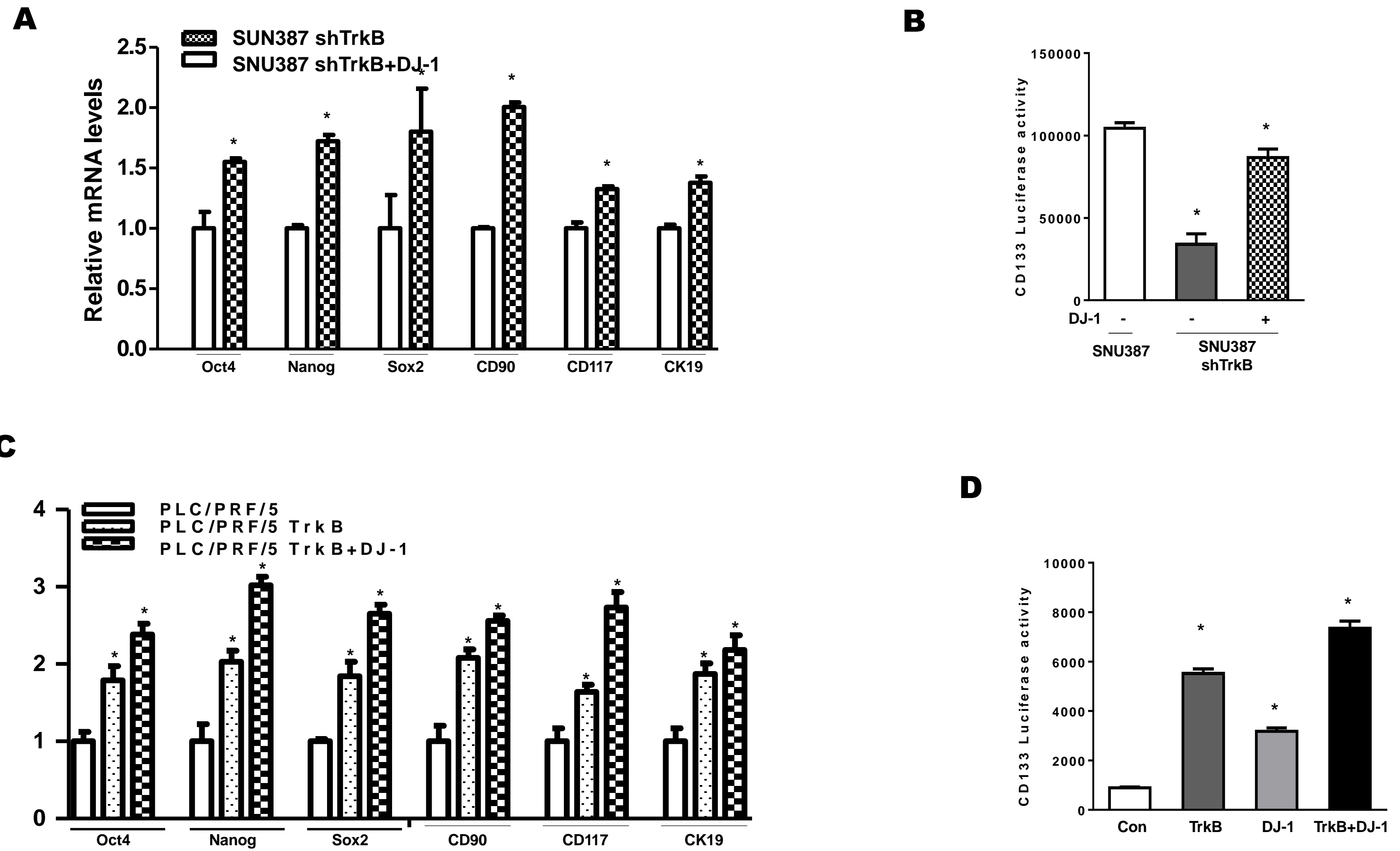

**A**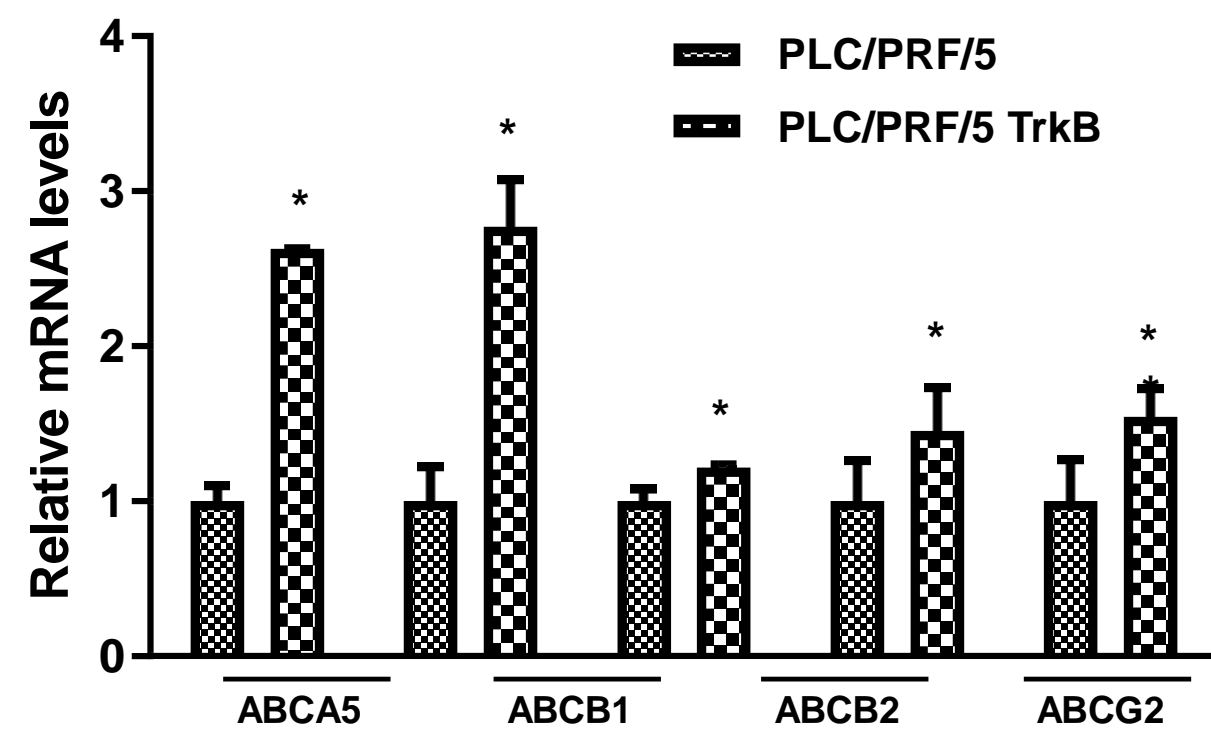**B**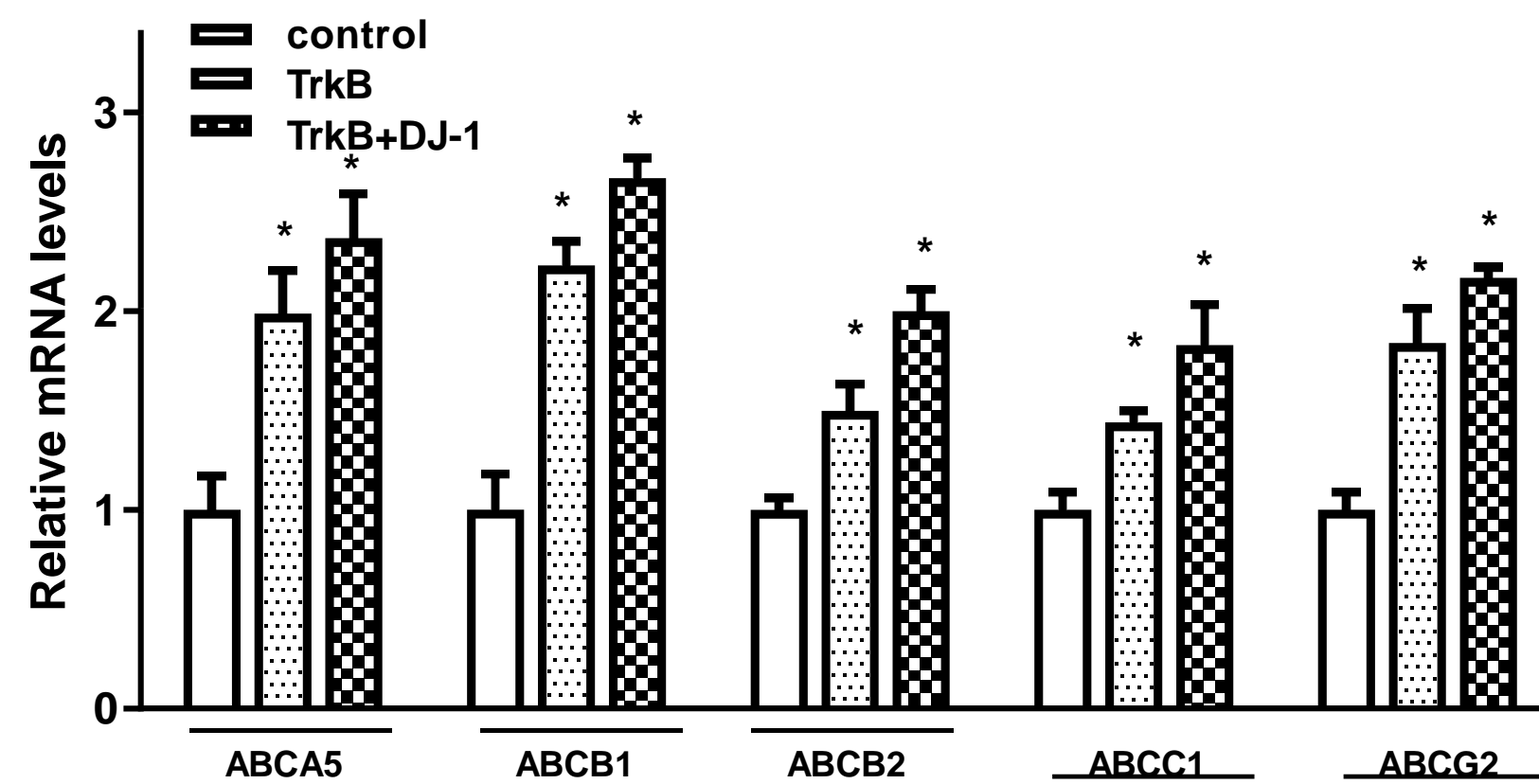

**A**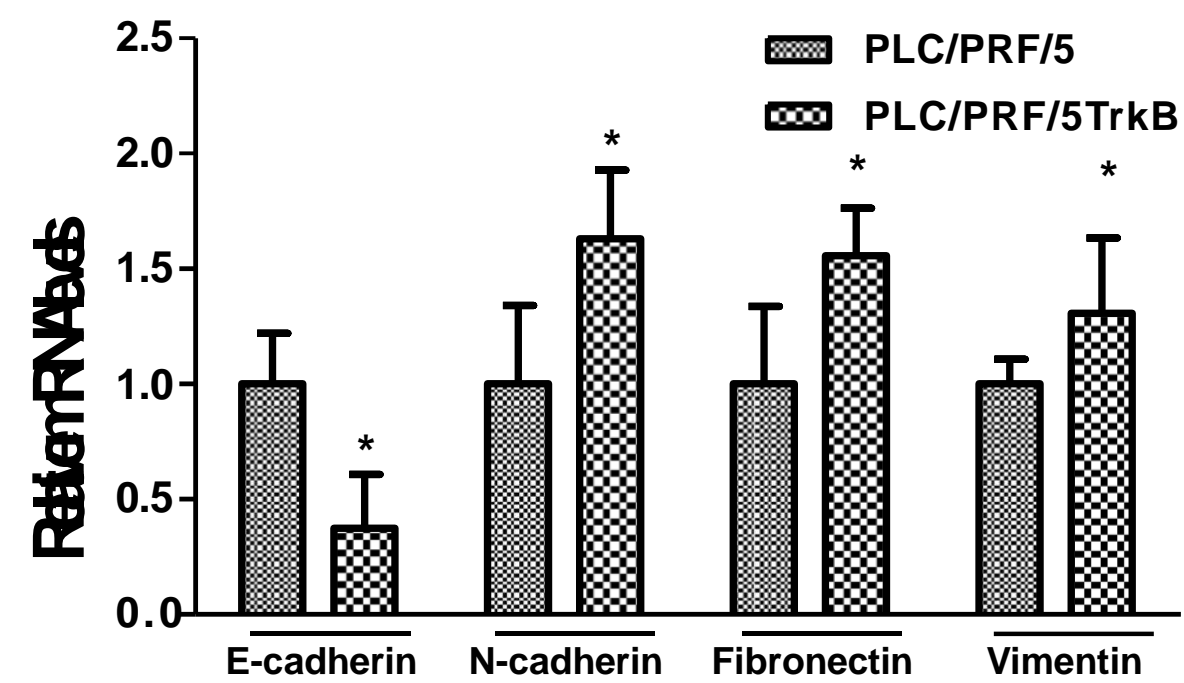**B**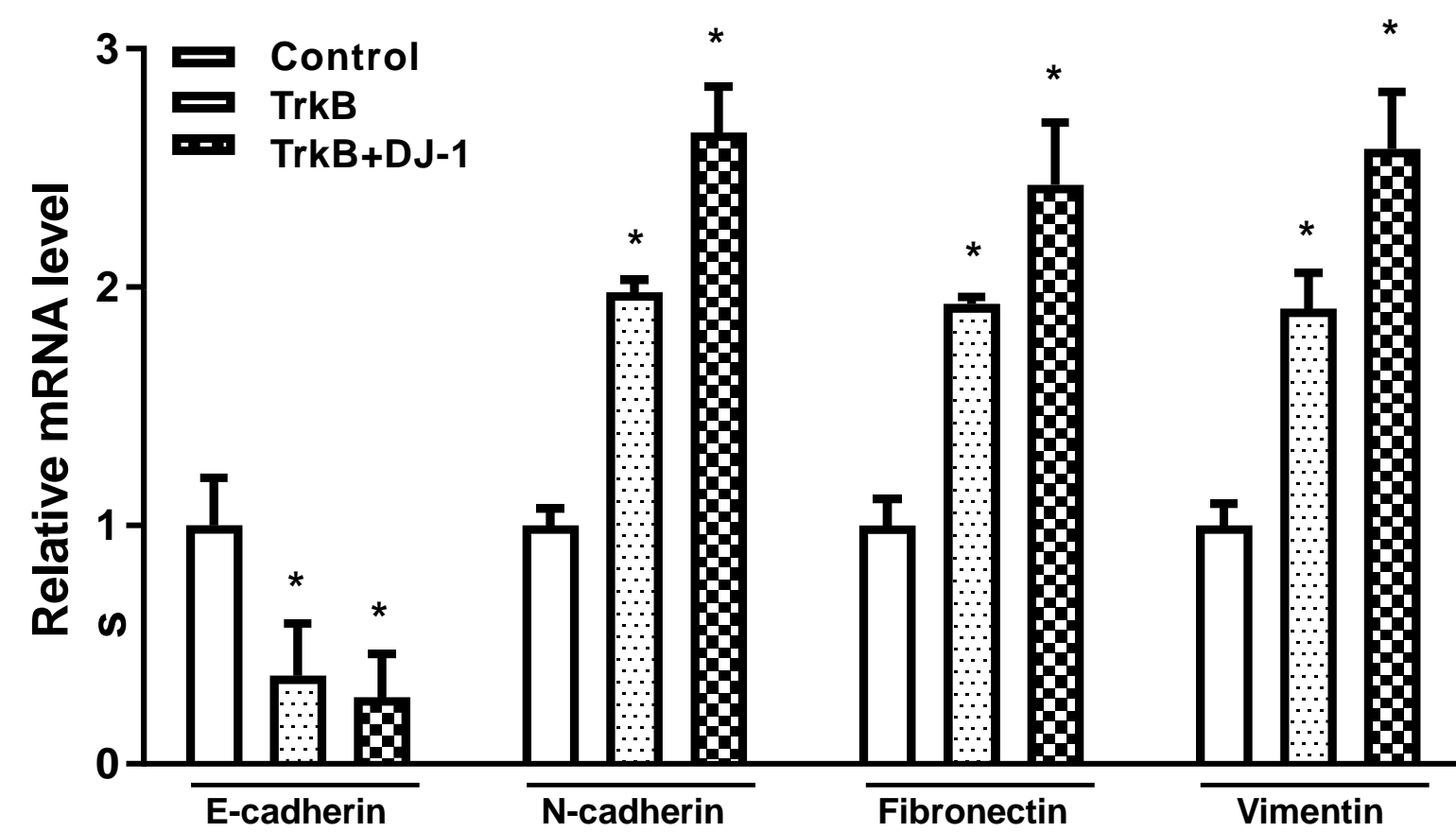

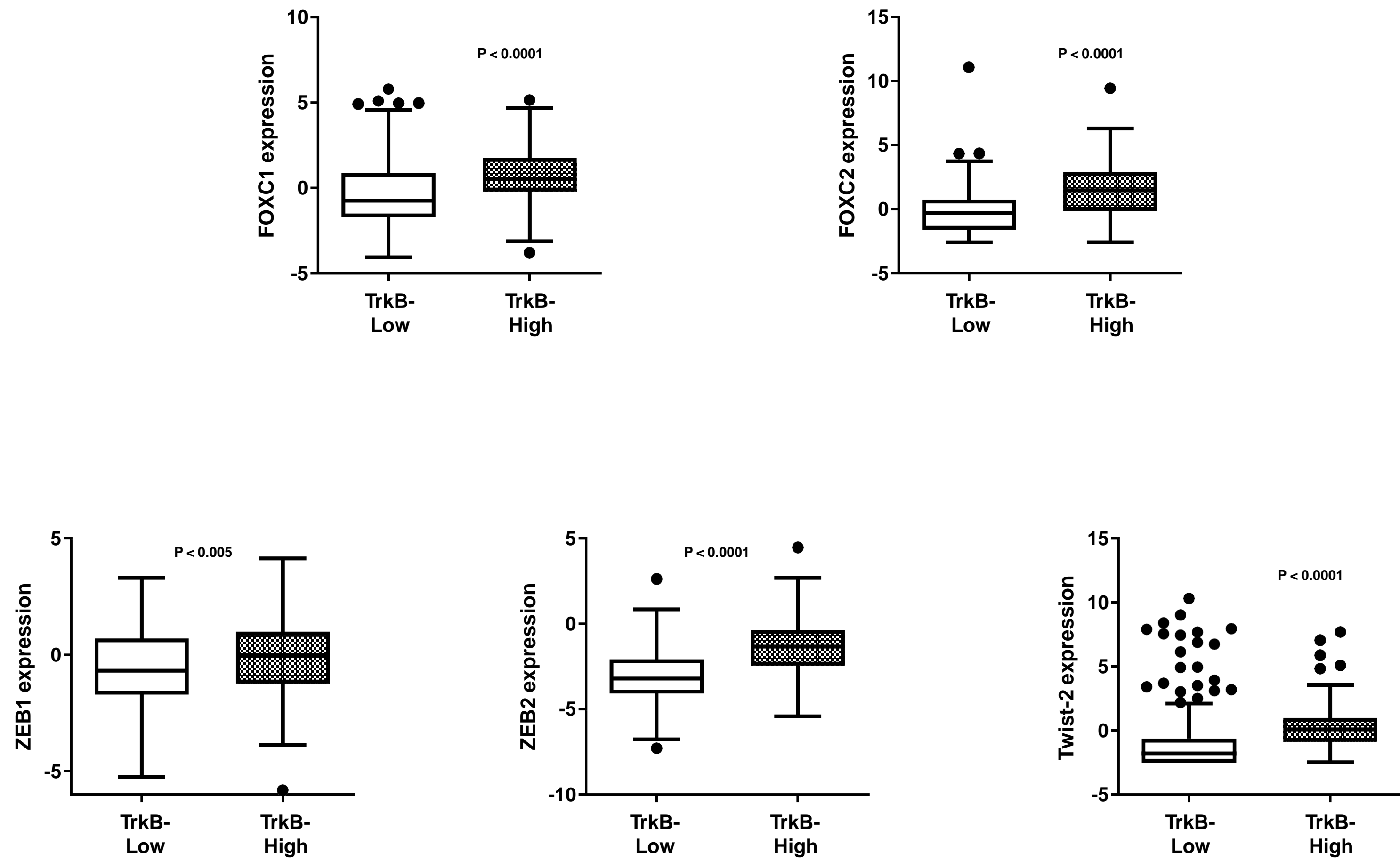

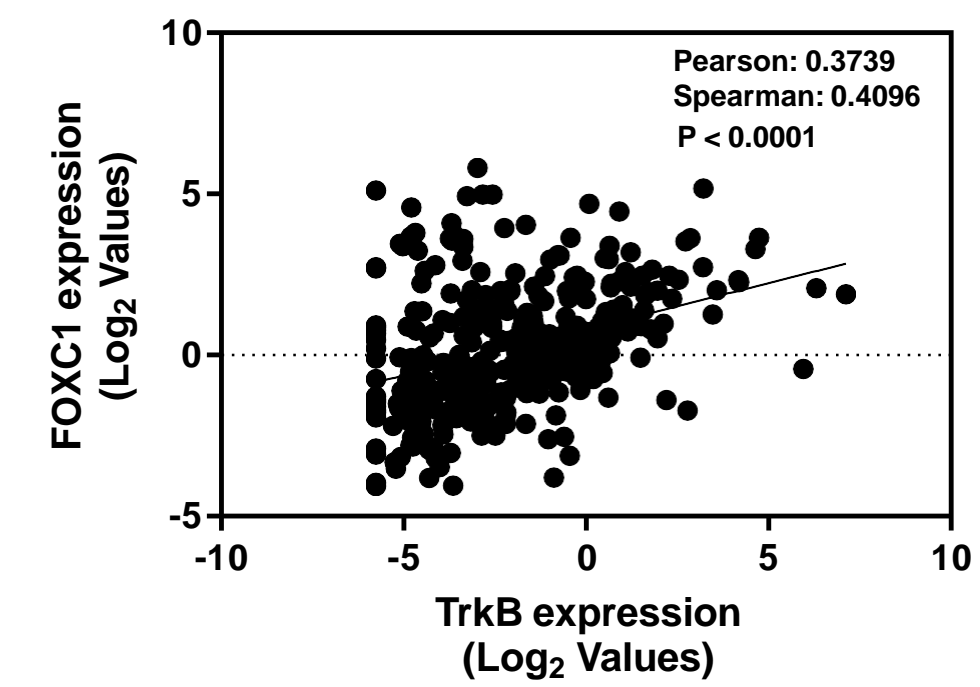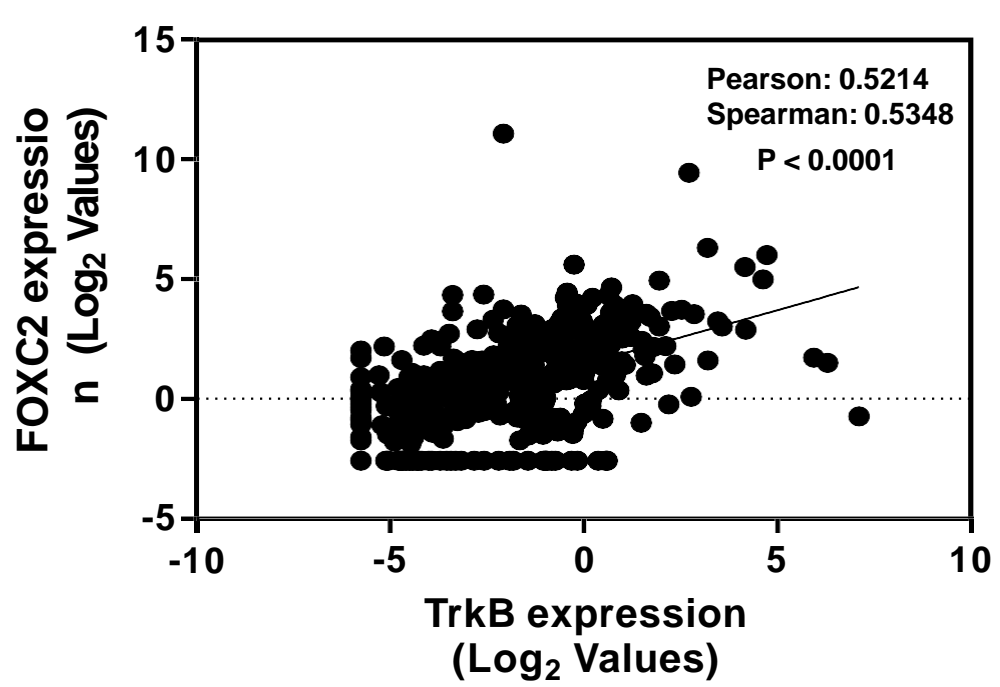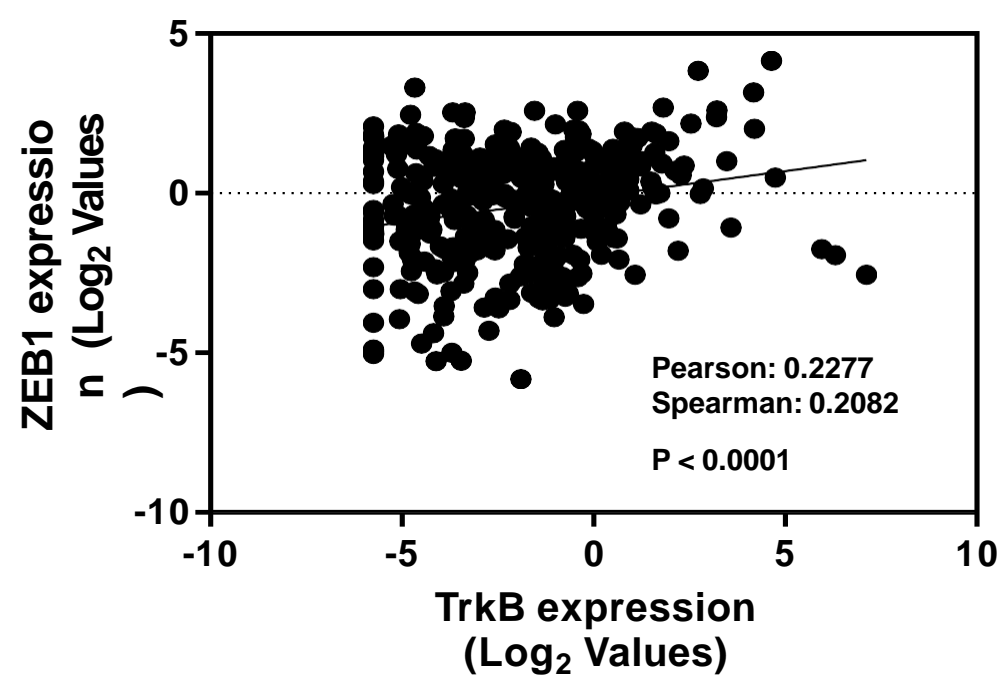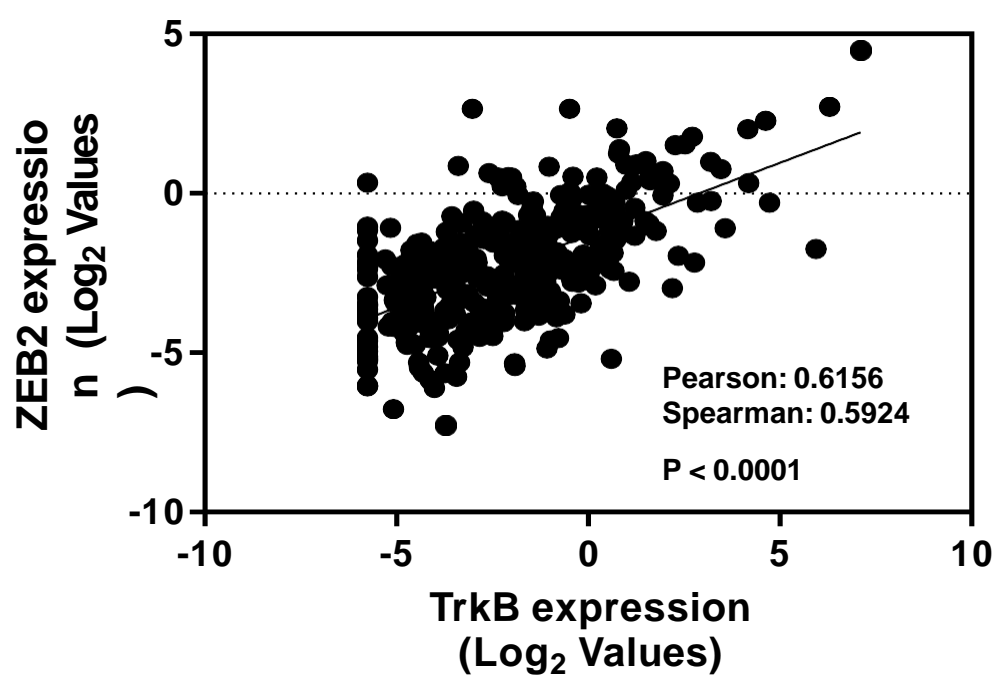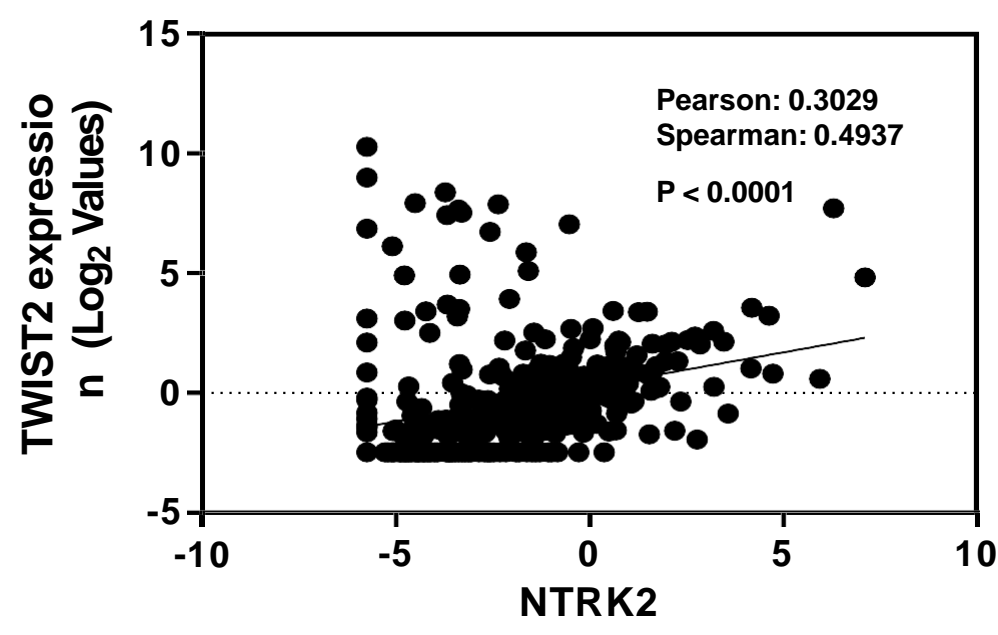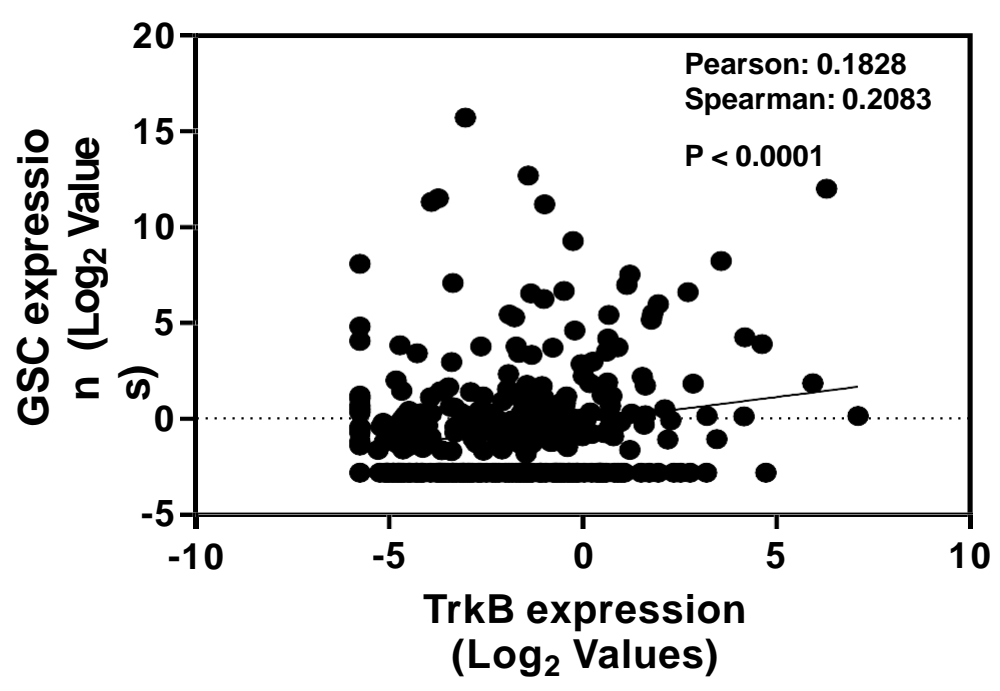

**A**

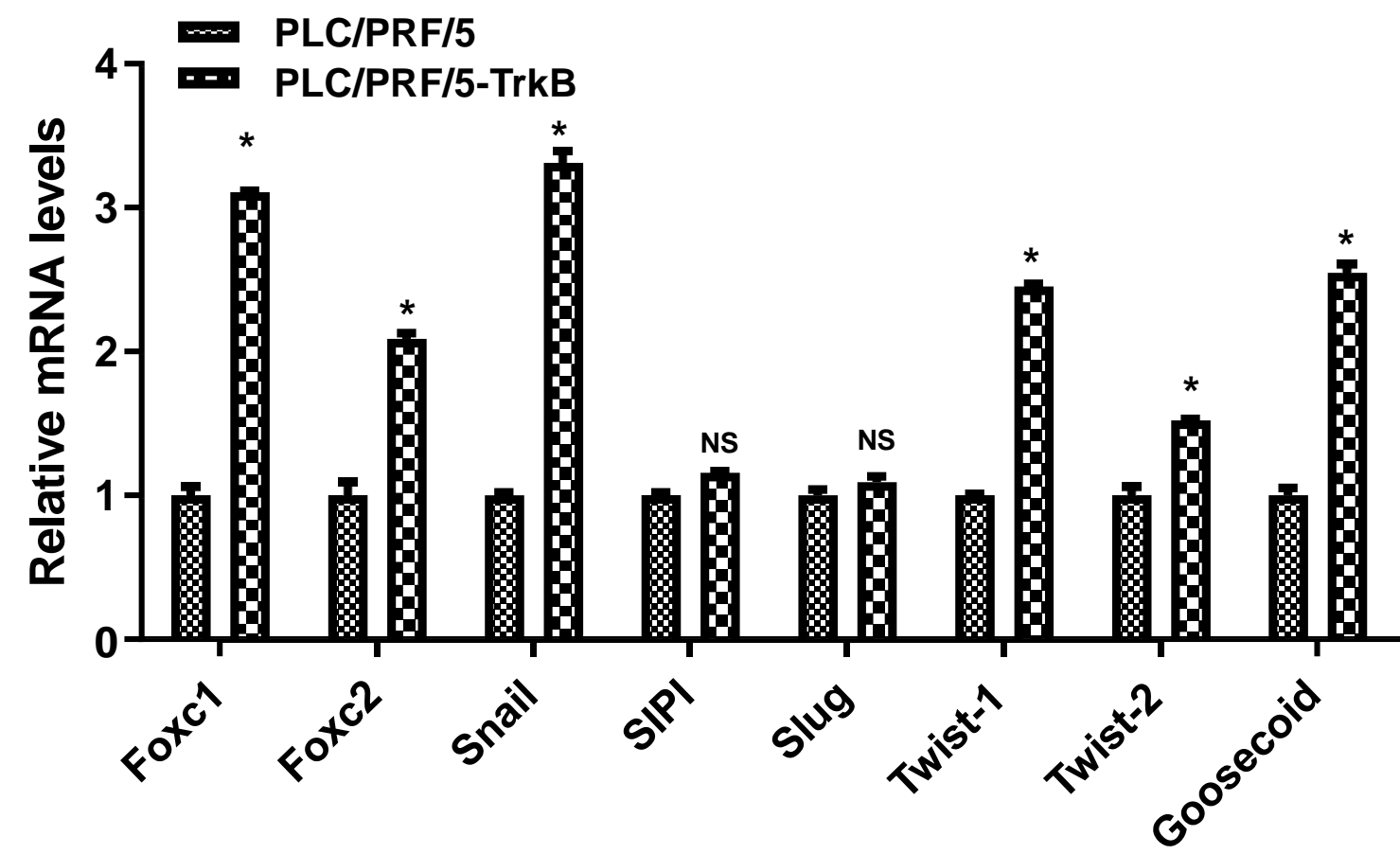

**B**

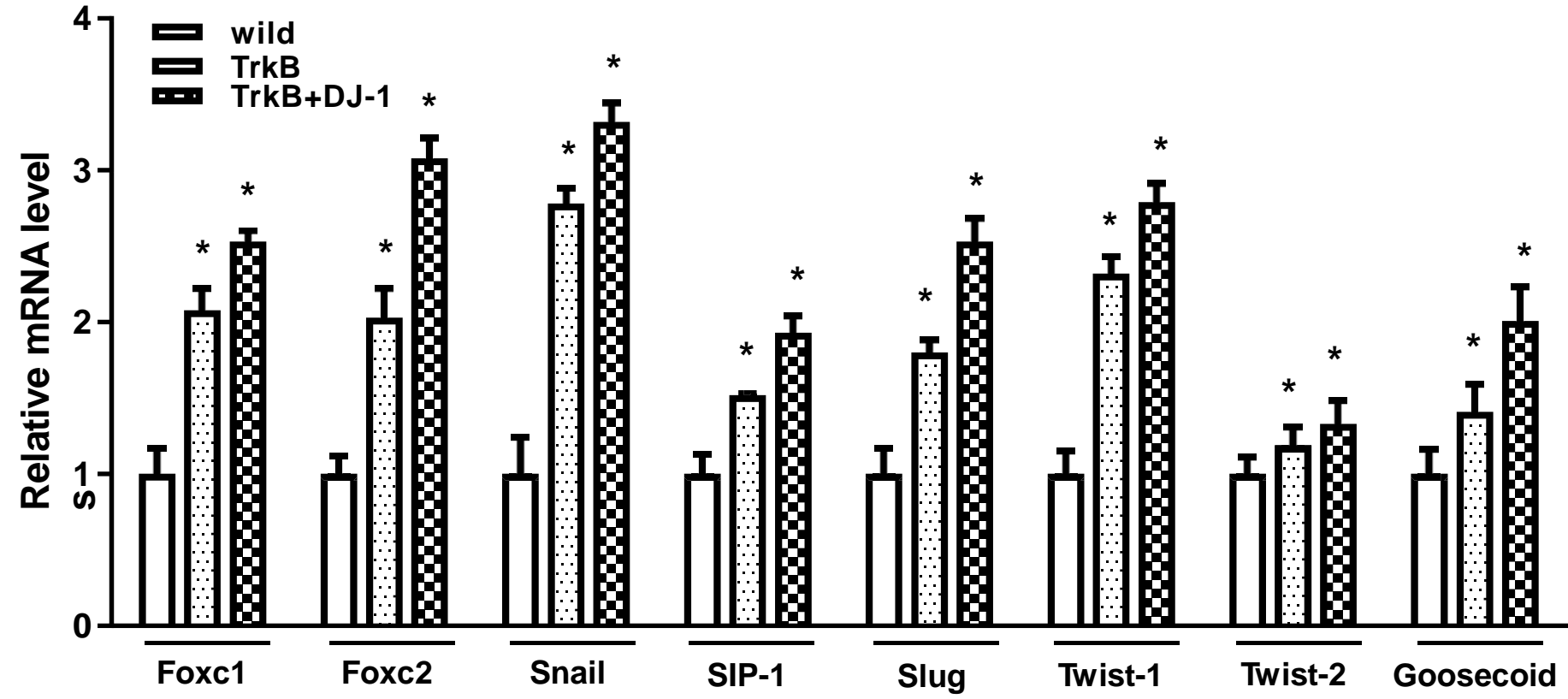

Supplement: Supplementary file 2 — Supplementary file2 (PDF 1255 KB) [file 18_2023_4960_MOESM2_ESM.pdf]
