# Supplementary material for: TrkB inhibition of DJ-1 degradation promotes the growth and maintenance of cancer stem cell characteristics in hepatocellular carcinoma
Source: Cell Mol Life Sci. 2023 Sep 25;80(10):303. doi: 10.1007/s00018-023-04960-z (PMC10520132; doi:10.1007/s00018-023-04960-z)
Supplement: Supplementary file 3 — Supplementary file3 (DOCX 21 KB) [file 18_2023_4960_MOESM3_ESM.docx]

**Supplemental Figure Legends**

**Figure S1.** The STAT3 and CD133 reporter gene constructs**.**

**Figure S2. Identification of TrkB expression on various hepatocellular carcinoma cells.** The real-time RT-PCR (qRT-PCR) analysis of TrkB expression in tumorigenic and metastatic human HCC cells. Data represent the mean of triplicate experiments ±SEM. P < 0.05 (*), *t*-test.

**Figure S3. The contribution of TrkB or DJ-1 in STAT3 expression.** The real-time RT-PCR (qRT-PCR) (A) and luciferase reporter (B) analysis of STAT3 expression in PLC/PRF/5 cells transfected with or without DJ-1 and TrkB. The data are presented as three independent experiments' mean ± SEM values.

**Figure S4. Identification of the complex formation of TrkB/STAT3 and DJ-1/STAT3 and DJ-1/TrkB in exogenous** **expression levels for transfected 293T cells.** (A) Exogenous expression levels for transfected 293T cells with the GFP-DJ-1 and Flag-STAT3 constructs. (B) Immunoblot analysis of the cell lysates and immunoprecipitates derived from 293T cells transfected with the V5-TrkB and Flag-STAT3 constructs. (C) Exogenous expression levels for transfected 293T cells with the GFP-DJ-1, Flag-STAT3 and Flag-STAT3 mt constructs. The primary antibodies used in the study were: anti-DJ-1 (1:1000; ab18257), anti-STAT3 (1:1000; #9139S), anti-V5 (1:1000; MA5-15253), anti-TrkB (1:1000 ab18987), anti-Flag (1:1000; F3165), anti-phospho-STAT3 (1:1000; #9145S), and anti-β-actin (1:1000; A1978). The cellular proteins were incubated with V5 (1:100; MA5-15253) primary antibody of volume of 2µL. Western blot analysis of the cell lysates from 293T cells.

**Figure S5. The expression level of native DJ-1 and GFP-tagged DJ-1 in the SNU387 and SNU387-shTrkB cells.** (A)The primary antibodies used in the study were: anti-DJ-1 (1:1000; ab18257), anti-phospho-STAT3 (1:1000; #9145S), anti-STAT3 (1:1000; #9139S), anti-TrkB (1:1000 ab18987), anti-GFP (1:1000; sc-9996), and anti-β-actin (1:1000; A1978). (B)Western blot analysis of the cell lysates from Chang, SNU387, and PLC/PRF/5 cells. The primary antibodies used in the study were: anti-DJ-1 (1:1000; ab18257), anti-STAT3 (1:1000; #9139S), anti-TrkB (1:1000 ab18987), and anti-β-actin (1:1000; A1978).

**Figure S6. The kinase activity of TrkB is required to activate STAT3 by induction of TrkB/DJ-1 interaction.** (A) Immunoblot analysis of the cell lysates and immunoprecipitates derived from 293T cells transfected with the V5-TrkB, V5 TrkB K588M, and Flag-STAT3 constructs, as indicated. (B) Immunoblot analysis of phospho-STAT3, STAT3, and TrkB expression in SNU387 TrkB-shRNA cells after transfected with TrkB and TrkB K588M. β-actin used as a loading control. The primary antibodies used in the study were: anti-STAT3 (1:1000; #9139S), anti-phospho-STAT3 (1:1000; #9145S), anti-TrkB (1:1000 ab18987), anti-GFP (1:1000; sc-9996), anti-V5 (1:1000; MA5-15253), anti-β-actin (1:1000; A1978) and anti-Flag (1:1000; F3165). The cellular proteins were incubated with V5 (1:100; MA5-15253) primary antibody of volume of 2µL.

**Figure S7. Inhibition of ubiquitination of DJ-1 by MG132 in 293T cell, PLC/PRF/5, and SNU387 cells.** Immunoblot analysis of the cell lysates and immunoprecipitates derived from (A)293T, (B)SNU387, and (C)PLC/PRF/5 cells. The primary antibodies used in the study were: anti-DJ-1 (1:1000; ab18257), anti-HA (1:1000; sc-7392), anti-TrkB (1:1000 ab18987), anti-GFP (1:1000; sc-9996), anti-V5 (1:1000; MA5-15253), and anti-β-actin (1:1000; A1978). The cellular proteins were incubated with anti-DJ-1 (1:100; ab18257) and anti-GFP (1:100; sc-9996) primary antibody of volume of 2µL. Cells were treated with 10uM MG132(M7449 sigma) for 12h.

**Figure S8. TrkB-mediated inhibition of DJ-1 degradation induces the proliferation of spheroid-forming cells.** The quantification and images of the spheroid formation of PLC/PRF/5 and PLC/PRF/5 TrkB cells. The data are presented as three independent experiments' mean ± SEM values, and representative results are shown. P < 0.05 (*), *t*-test.

**Figure S9. TrkB-mediated inhibition of DJ-1 degradation significantly enhances the transition of CSCs.** (A) Relative mRNA expression of human embryonic stem (hES) cells markers (Oct4, Nanog, Sox2) and specific CSC markers (CD90, CD117, CD133, and CK19) of HCC in PLC/PRF/5 and PLC/PRF/5 TrkB cells. P < 0.05 (*), *t*-test. (B) The Luciferase reporter assay for transcriptional activity of CD133 in PLC/PRF/5 and PLC/PRF/5 TrkB cells or SNU387 control-shRNA and SNU387 TrkB-shRNA cells. Each bar represents the mean ± SEM of three experiments. *P* < 0.05, *t*-test. (C) Box-and-whisker (Tukey) plots of CD117, CK19, and CD90 expression in human HCC patients between high or low TrkB expression. The average expression value of TrkB from the TCGA datasets was determined, rank-ordered, and then divided into two equal groups(N=372). P < 0.05, 0.005, or 0.0001 relative to TrkB-Low; *t*-test. (D) The correlation between TrkB and CD133, CD117, CK19, or CD90 expression in TCGA datasets(N=372). P < 0.0001; Spearman's correlation coefficient. All the experiments were performed in triplicates, and representative results are shown.

**Figure S10. Upregulation of TrkB and DJ-1 is required for the induction of CD133 expression.** (A) Relative mRNA expression of hES cells markers and specific CSC markers of HCC in SNU387 TrkB-shRNA cells transfected with DJ-1. P < 0.05 (*), *t*-test. (B) The Luciferase reporter assay for transcriptional activity of CD133 in SNU387 control-shRNA or SNU387 TrkB-shRNA cells transfected with DJ-1. Each bar represents the mean ± SEM of three experiments. *P* < 0.05, *t*-test. (C) Relative mRNA expression of hES cell markers and specific CSC markers of HCC in PLC/PRF/5 cells transfected with DJ-1 or TrkB. P < 0.05 (*), *t*-test. (D) The Luciferase reporter assay for transcriptional activity of CD133 in PLC/PRF/5 cells transfected with DJ-1 or TrkB. P < 0.001 (*), *t*-test. All the experiments were performed in triplicates, and representative results are shown.

**Figure S11. TrkB-mediated inhibition of DJ-1 degradation induces the expression of ABC transporters.** (A) Relative mRNA expression of human ABC transporters (ABCA5, ABCB1, ABCB2, and ABCG2) in PLC/PRF/5 and PLC/PRF/5 TrkB cells. P < 0.05 (*), *t*-test. (B) Relative mRNA expression of human ABC transporters in PLC/PRF/5 cells with or without TrkB or DJ-1. P < 0.05 (*), *t*-test. All the experiments were performed in triplicates, and representative results are shown.

**Figure S12. Effects of TrkB and DJ-1 in EMT progression.** (A) Relative mRNA expression encoding fibronectin, vimentin, E- and N-cadherin in PLC/PRF/5 or PLC/PRF/5 TrkB cells. P < 0.05 (*), *t*-test. (B) Relative mRNA expression of epithelial and mesenchymal markers in PLC/PRF/5 cells transfected with TrkB or DJ-1. P < 0.05 (*), *t*-test. All the experiments were performed in triplicates, and representative results are shown.

**Figure S13. Upregulation of TrkB induces EMT-TFs expression.** Box-and-whisker (Tukey) plots of expression of EMT-TFs, including FOXC1, FOXC2, ZEB1, ZEB2, and Twist-2 in human HCC patients between high or low TrkB expression. The average expression value of TrkB from the TCGA datasets was determined, rank-ordered, and then divided into two equal groups. P < 0.05 relative to TrkB-Low; *t*-test(N=372). All the experiments were performed in triplicates, and representative results are shown.

**Figure S14. The correlation between TrkB and EMT-TFs expression.** P < 0.0001; spearman or Pearson's correlation coefficient(N=372).

**Figure S15. TrkB-mediated inhibition of DJ-1 degradation required for the induction of EMT-TFs.** (A) Relative mRNA expression of EMT-TFs, Foxc1, Foxc2, Snail, SIP1, Slug, Twist-1, Twist-2, and Goosecoid in PLC/PRF/5 or PLC/PRF/5 TrkB cells. P < 0.05 (*); NS: not significant; *t*-test. (*), *t*-test. (B) Relative mRNA expression of EMT-TFs in PLC/PRF/5 cells transfected with TrkB or DJ-1. P < 0.05 (*), *t*-test. All the experiments were performed in triplicates, and representative results are shown.
